# Removing Batch Effects from Longitudinal Gene Expression - Quantile Normalization Plus ComBat as Best Approach for Microarray Transcriptome Data

**DOI:** 10.1371/journal.pone.0156594

**Published:** 2016-06-07

**Authors:** Christian Müller, Arne Schillert, Caroline Röthemeier, David-Alexandre Trégouët, Carole Proust, Harald Binder, Norbert Pfeiffer, Manfred Beutel, Karl J. Lackner, Renate B. Schnabel, Laurence Tiret, Philipp S. Wild, Stefan Blankenberg, Tanja Zeller, Andreas Ziegler

**Affiliations:** 1 Clinic for General and Interventional Cardiology, University Heart Center Hamburg, Hamburg, 20246, Germany; 2 Institute of Medical Biometry and Statistics, University Medical Center Schleswig-Holstein, Campus Luebeck, Lübeck, 23562, Germany; 3 German Center for Cardiovascular Research (DZHK e.V.), partner site Hamburg, Lübeck, Kiel, 20246, Germany; 4 Institut National de la Santé et de la Recherche Médicale (INSERM), Unité Mixte de Recherche(UMR) enSanté 1166, F-75013, Paris, France; 5 Institute for Cardiometabolism and Nutrition (ICAN), F-75013 Paris, France; 6 Sorbonne Universités, Université Pierre et Marie Curie (UPMC Univ Paris 06), UMR_S1166, Team Genomics & Pathophysiology of Cardiovascular Diseases, F-75013, Paris, France; 7 Institute of Medical Biostatistics, Epidemiology and Informatics (IMBEI) at the University Medical Center of the Johannes Gutenberg University Mainz, Mainz, 55131, Germany; 8 Experimental Ophthalmology, Department of Ophthalmology, University Medical Center of the Johannes Gutenberg University, Mainz, 55131, Germany; 9 Department of Psychosomatic Medicine and Psychotherapy, University Medical Center of the Johannes Gutenberg-University Mainz, Mainz, Germany; 10 Institute for Clinical Chemistry and Laboratory Medicine, University Medical Center Mainz, Mainz, Germany; 11 Preventive Cardiology and Preventive Medicine, Center for Cardiology, University Medical Center Mainz, Mainz, 55131, Germany; 12 Center for Thrombosis and Hemostasis, University Medical Center Mainz, Mainz, 55131, Germany; 13 German Center for Cardiovascular Research (DZHK e.V.), partner site Rhine Main, Mainz, 55131, Germany; 14 Center for Clinical Trials, University of Lübeck, Lübeck, 23562, Germany; University Medicine Greifswald, GERMANY

## Abstract

Technical variation plays an important role in microarray-based gene expression studies, and batch effects explain a large proportion of this noise. It is therefore mandatory to eliminate technical variation while maintaining biological variability. Several strategies have been proposed for the removal of batch effects, although they have not been evaluated in large-scale longitudinal gene expression data. In this study, we aimed at identifying a suitable method for batch effect removal in a large study of microarray-based longitudinal gene expression. Monocytic gene expression was measured in 1092 participants of the Gutenberg Health Study at baseline and 5-year follow up. Replicates of selected samples were measured at both time points to identify technical variability. Deming regression, Passing-Bablok regression, linear mixed models, non-linear models as well as *ReplicateRUV* and *ComBat* were applied to eliminate batch effects between replicates. In a second step, quantile normalization prior to batch effect correction was performed for each method. Technical variation between batches was evaluated by principal component analysis. Associations between body mass index and transcriptomes were calculated before and after batch removal. Results from association analyses were compared to evaluate maintenance of biological variability. Quantile normalization, separately performed in each batch, combined with *ComBat* successfully reduced batch effects and maintained biological variability. *ReplicateRUV* performed perfectly in the replicate data subset of the study, but failed when applied to all samples. All other methods did not substantially reduce batch effects in the replicate data subset. Quantile normalization plus *ComBat* appears to be a valuable approach for batch correction in longitudinal gene expression data.

## Introduction

Gene expression profiles measured by microarrays are subject to variations caused by biological and technical effects. In a transcriptome study, systematic differences resulting from biological conditions are of interest, whereas technical variation should be minimal. The highest proportion of technical variation is systematic and potentially introduced by the RNA processing steps [1]. In addition, RNA quality and sample storage time influence overall variation of transcriptomes [2]. Therefore, it is mandatory to avoid batch effects wherever possible and to set up a suitable strategy for technical noise reduction after mRNA quantification.

In the ideal experimental setting, all samples would be processed in a single batch. However, caused by technical limitations for the number of samples that can be processed at once, this is impossible when large sample sets are processed. For instance, RNA isolation can only be performed for a small number of samples in parallel. Amplification and labeling of RNA is usually carried out on well plates of 96 or 384 samples, and several of these plates are required for a large-scale transcriptome study. In addition, batch sizes of the scanning step are currently limited to 48 samples on the Affymetrix platform and 172 samples for Illumina. RNA quality, sample storage time and plate layout are important additional technical factors influencing the association analysis of gene expression data and common disease risk factors [2]. Consequently, batch effects cannot be avoided in studies comprising a large number of subjects, and removal of these effects is necessary for reliable differential expression analysis.

Technical factors, including batch effects, also affect longitudinal gene expression analysis. As RNA is collected at different time points in these studies, additional factors possibly influencing gene expression levels need to be considered. Depending on the time between measurements, the biochemistry of the assays, the scanning device and even the microarray technology may have changed. If samples from different time points are processed in parallel, storage time of samples might affect gene expression levels, leading to batch effects. Consequently, it is mandatory to reduce batch effects without eliminating biological variation and to demonstrate repeatability of gene expression levels.

Several approaches have been proposed for batch effect removal from gene expression data [3–7]. Linear models can be applied to estimate batch effects between technical replicates measured at each time point. Resulting effect estimates can then be utilized for correcting overall gene expression levels between batches. An alternative regression approach is Deming regression [8], which allows to model normally distributed errors independently for two measurement methods. Passing-Bablok regression also models errors independently [9], but this approach does not introduce assumptions about the underlying error distributions. Workman and colleagues [10] reported that linear models are not capable to fully correct for batch effects and proposed the non-linear method *qspline*. *qspline* integrates quantile information of gene expression distributions and uses a cubic spline to fit all values dependent on signal intensities. *ComBat* combines location and scale adjustment with empirical Bayes to remove batch effects [11]. Location and scale parameters, representing mean and variance, are estimated for each batch and each gene independently. Batch effects are estimated by empirical Bayes and used for batch effect removal. *ComBat* has been successfully applied to several datasets [3, 12–14], and using a single reference sample for each batch, its usefulness has been demonstrated for cross-sectional data [13]. The aforementioned approaches assume that the batches are known. Different matrix factorization-based methods were developed for the case that unwanted factors of variation are unknown, e.g. surrogate variable analysis (SVA) [15] or removal of unwanted variation (RUV) [16]. On microarrays, background noise is often modelled by negative control genes. These should not be differentially expressed between biological conditions. In contrast, observed differences between negative control genes can be considered as technical variation. RUV utilizes negative controls combined with technical replicates when estimating and correcting for batch effects (*ReplicateRUV*) [17]. So far, none of these methods for batch effect removal has been evaluated in large-scale longitudinal gene expression studies.

The aim of this study was to identify the best method to remove batch effects in large-scale longitudinal gene expression data. Seven different approaches were applied to gene expression data sets consisting of 1092 individuals from the Gutenberg Health Study (GHS) available at baseline and the 5-year follow up visit.

## Materials and Methods

### Study description

Study participants of both sexes aged between 35–74 years, were included into the Gutenberg Health Study (GHS), a community-based, prospective, observational single-center cohort study in the Rhine-Main region in Western Mid-Germany. All subjects provided written informed consent. The study was approved by the local ethics committee (Ethik-Kommission—Landesärztekammer Rheinland-Pfalz) and by the local and federal data safety commissioners.

### Monocytes enrichment and RNA isolation

For enrichment of monocytes and RNA isolation, the same methodological approach was used at both time points [18]. In brief, for monocytes enrichment the RosetteSep Monocyte Enrichment Cocktail (StemCell Technologies, Vancouver, Canada) was used directly after blood sampling. Total RNA was extracted on the same day of blood sampling using Trizol extraction (Invitrogen/Thermo Fisher, Darmstadt, Germany) and purification by the RNeasy Mini Kit (Qiagen, Hilden, Germany). Monocyte enrichment and RNA isolation were performed in the GHS study center by the same personnel for both baseline (BL) and follow up (FU) visit. The integrity of the total RNA was assessed through analysis on an Agilent Bioanalyzer 2100 (Agilent Technologies, Böblingen, Germany). Samples with a RNA integrity number (RIN) <7 were excluded. Total RNA was stored at –80°C until further processing; Time of storage was 2–14 month at both time points with the same time span for RNA samples of the same individuals at BL and FU.

### RNA processing and microarray hybridization

**Fig 1:**
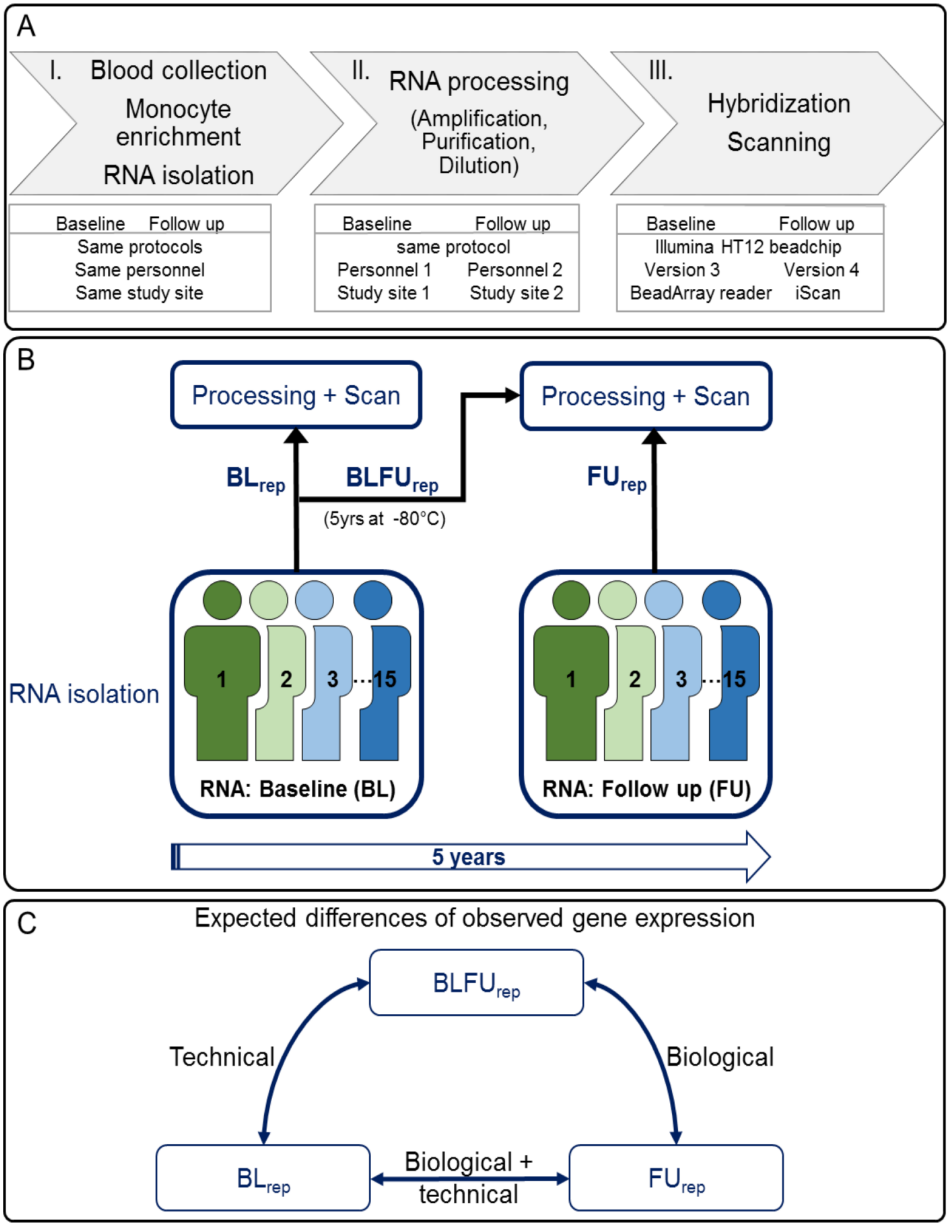
Experimental setting. A: Work flow of RNA extraction, processing and hybridization at study’s baseline (BL) and follow up (FU) examination. I) Blood collection, monocyte enrichment and RNA isolation were performed following the same standard operating procedures at the study center for both, BL and FU. II) RNA processing including amplification, purification, and dilution was performed using the same protocol at BL and FU, however, these steps were performed by different personnel a different study site. III) At BL, the Illumina HT12 BeadChips version 3 was used for hybridization and at FU the Illumina HT12 BeadChips version 4. At BL, the BeadArray Reader was used for the scan of the beadchips and at FU, the iScan was used. B: Definition of replicated samples. 15 subjects were randomly selected. At BL and FU, RNA was isolated from these 15 subjects. Based on the time point of RNA isolation and the time point of RNA processing, hybridization (preparation) and scan, three groups of sample replicates were defined: i) RNA isolated, prepared and arrays scanned at BL (BL_rep_), ii) RNA isolated at BL, stored for 5 years at -80°C, then prepared and arrays scanned at FU (BLFU_rep_) and iii) RNA isolated, prepared and scanned at FU (FU_rep_). C: Factors affecting observed gene expression differences between replicate measures. BL_rep_ and BLFU_rep_ differ by the time point of RNA preparation and array scan and thus reflect technical differences. For BLFU_rep_ and FU_rep_, RNA preparation and array scan were performed at the same time point, therefore, observed variation mainly reflects biological differences. Observed differences between BL_rep_ and FU_rep_ comprise technical and biological variation. (rep = replicated sample).

Different steps of the work flow were harmonized at baseline and follow up. Fig 1A illustrates blood sampling, RNA preparation and processing as well as hybridization and scanning of RNA samples.

RNA processing for gene expression analysis has been described before [18]. In brief, 200 ng of total RNA was amplified and biotinylated using the Illumina TotalPrep-96 RNA Amplification Kit (Ambion, Darmstadt, Germany). At baseline, cDNA and cRNA were automatically purified using the MagMax Express96 magnetic particle processor (Applied Biosystems, Waltham, MA, USA). At the follow up time point, cDNA and cRNA were automatically purified using the Agilent Bravo system (Agilent, Santa Clara, CA, USA). Concentration of cRNA was determined on a Tecan InfiniTE M200 (Tecan, Zürich, Switzerland) at both time points. Dilution of each cRNA sample to 140ng/μl was performed automatically with the Tecan Freedom EVOlyzer (BL) or the Agilent Bravo system (FU).

For hybridization onto microarrays, 700 ng of purified and diluted cRNA were used at both time points. At BL, cRNA was hybridized to the Human HT-12 v3 BeadChip (Illumina) at 58°C for 16-18h. After hybridization, BeadChips were washed and stained according to the manufacture´s instruction. BeadChips were scanned using Illuminas´ Bead Array Reader. At FU, cRNA was hybridized to the Human HT-12 v4 BeadChip (Illumina) at 58°C for 16-18h. After hybridization, BeadChips were washed and stained according to the manufactures instruction. BeadChips were scanned using the Illumina iScan.

### Replicates of RNA samples

To distinguish batch effects from biological differences between baseline visit (BL) and 5-year follow up (FU), replicates of RNA samples were measured as illustrated in Fig 1B and 1C. To this end, 15 GHS participants were randomly selected and duplicates or triplicates of RNA isolated at each time point were measured as depicted in Table 1. In total, 132 measures were used, which are assigned to three different groups:

1. BL_rep_: 35 measurements of RNA isolated from the 15 individuals, processed, hybridized and scanned only at BL as duplicates or triplicates.
2. BLFU_rep_: 43 measurements of RNA isolated from the 15 individuals at BL, and processed, hybridized and scanned as duplicates or triplicates repeatedly at FU.
3. FU_rep_: 54 measurements of RNA isolated from the 15 individuals at FU and processed, hybridized and scanned only at FU.

**Table 1:** Number of RNA measurements used for evaluating batch effect removal approaches at baseline and 5-year follow up.

	RNA isolated at			
	Baseline		5-year follow up	
	RNA hybridized and scanned at		RNA hybridized and scanned at	
Subject	Baseline (BL_rep_^a^)	Baseline and 5-year follow up (BLFU_rep_^b^)	5-year follow up (FU_rep_^c^)	Sum of measurements
1	2	3	3	8
2	2	3	3	8
3	3	2	3	8
4	2	3	4	9
5	2	3	4	9
6	2	3	4	9
7	2	3	4	9
8	2	3	4	9
9	2	3	4	9
10	2	2	4	8
11	3	3	4	10
12	3	3	2	8
13	2	3	4	9
14	3	3	4	10
15	3	3	3	9
**Sum of measurements**	**35**	**43**	**54**	**132**

RNA samples of 15 distinct individuals were extracted at baseline and 5-year follow up. Samples extracted at baseline were processed, hybridized and scanned in duplicates or triplicates at baseline and follow up. Different overall gene expression between time points for those repeated measures reflect batch effects, because respective RNA samples origin from the same time point. Hence, those groups were used to assess batch effects and evaluate batch effect removal methods. Samples isolated at follow up were processed, hybridized and scanned in duplicates or triplicates at follow up only. Differences between those measurements and replicates isolated and measured at baseline can be explained by technical and biological differences. For each individual the number of measurements according to the different groups is given in this table.

^a^BL_rep_: RNA was isolated, processed, hybridized and scanned at baseline visit.

^b^BLFU_rep_: RNA was isolated at baseline and processed, hybridized and scanned at follow up.

^c^FU_rep_: RNA was isolated, processed, hybridized and scanned at the 5-year follow up.

Observed overall gene expression differences between BL_rep_ and BLFU_rep_ are largely due to batch effects, whereas differences between BLFU_rep_ and FU_rep_ mainly reflect biological differences between examination dates (Fig 1C). Observed differential expression between BL_rep_ and FU_rep_ comprise both, biological variation and batch effects. These properties were utilized to evaluate removed technical and preserved biological variation after applying batch effect removal strategies.

### Data pre-processing and QC

#### Pre-processing

Microarray data of both time points was read into the R environment using the R/Bioconductor package *beadarray* [19]. To process Illumina iScan data from FU visit, they had to be converted using the *beadarray* function *processSwathData*. In total, 1251 arrays were available for BL and 1266 for FU with hybridized RNA from both examination dates. The signal to noise ratio (SNR), defined as the 95^th^ percentile divided by the 5^th^ percentile of all expression values for one array, was used as sample quality filter. Only microarrays with an SNR>6 were kept for further analysis. Within each batch, mean log_2_-transformed gene expression was calculated for each sample, and a quality filter of mean expression levels ± 3 standard deviations was applied. None of the samples were excluded based on these filters.

#### Shared probes between chip versions

Illumina HT12 version 3 arrays used at BL visit, contained 48,803 probes on each chip. For HT12 version 4, which was used 5 years later, 47,231 probes were available. 39,426 probes with 100% sequence identity were shared between both Illumina HT12 versions. Before performing further pre-processing steps, all probes that were disjunctive between chip versions were removed. Only these probes were used for all further pre-processing steps.

#### Removal of mixed-up samples

Two strategies were applied to identify and remove mixed-up samples based on their gene expression differences between BL and FU. In a first step, sex-specific gene expression was utilized to identify samples with changed expression between time points. Therefore, differences of X inactive specific transcript (*XIST*, expressed in females) and eukaryotic translation initiation factor 1A, Y-linked (*EIF1AY*, expressed in males) mRNA between BL and FU were assessed. In total, 35 sample pairs with expression changes more than 3 standard deviations from the mean differences were excluded. In a second step, the polymorphism-in-probe problem [20] was utilized given the fact that single nucleotide polymorphisms (SNPs) do not change over time. For this purpose, SNP rs8676 located in the Illumina probe ILMN_2399463 was selected based on its high minor allele frequency (MAF = 47%) from Ramasamy et al [20]. Mean expression of ILMN_2399463 was highly dependent on rs8676 alleles (AA: = 6.5, AG: = 8.8, GG: = 9.9). Nineteen sample pairs with strong ILMN_2399463 expression changes between BL and FU (> 3 standard deviations from the mean difference) were identified, of which 8 were already identified based on sex-specific signatures in the first step. After excluding potentially mixed-up samples, 1092 samples with gene expression data available at BL and FU were kept for further analysis.

#### Filtering expressed probes

Illumina *GenomeStudio* built-in function for detecting expressed probes was compared with the corresponding function from *beadarray* R package [19]. Both methods use negative control probes to consider background noise. Because *beadarray* method *Detect* was more robust to outliers of the intensity distribution of negative controls, it was used for expression detection calling. Each probe with a detection above background (DABG) p-value < 0.05 was marked as detected for one individual. No sample met the exclusion criteria of < 6000 detected probes. For further analyses, 20,399 probes were kept which were called expressed in > 10% of all BL and > 10% of all FU samples. Expression values for all samples were summarized using *beadarray summarize* function, log_2_ transformed and transformed into objects accessible by R/Bioconductor package *lumi* [21].

### Assessment of batch effects on gene expression data

Distributions and variance of overall gene expression were evaluated in different ways. Density plots were created using the *lumi* method *plotDensity* and assigned to batches by different colors. For comparing distributions of probe expression between BL replicates measured at different time points, boxplots were used. Principal component analyses (PCA) were performed by applying R function *prcomp* to separate overall variance into independent components. Principal components reflect different sources of technical or biological variability. The first few principal components explain the highest proportion of overall gene expression variance: by plotting principal component 2 against principal component 1 potential batch effects can be visualized. Overall gene expression was hierarchically clustered based on pairwise Euclidean distances between samples using R function *hclust* and plotted in a dendrogram to visualize clustering of samples into batches.

### Methods used for batch adjustment

Seven methods for batch effect removal were compared: (i) Deming regression, (ii) Passing-Bablok regression, iii) linear mixed models, iv) third order polynomial regression, v) *qspline*, vi) *ComBat [11]* and vii) *ReplicateRUV[17]*. For i)–iv), differences in gene expression between BL_rep_ and BLFU_rep_ measurements were estimated by the particular regression model. Effect estimates were then used to correct batch effects in BL_rep_ measurements by rescaling gene expression levels of all probes. For methods v), vi) and vii), R/Bioconductor packages *affy* [22], *sva [5]* and *RUVnormalize* [17] were applied to all BL_rep_ and BLFU_rep_ measurements. Different linear regression models were applied to estimate a linear equation, which was subsequently used to rescale gene expression data of BL_rep_ measurements. The set, in which correction parameters were estimated, slightly differed between the models as described in the following.

#### Deming regression

For Deming regression [8], R package *mcr* was used to estimate parameters for fitting BL to FU expression values. All pairs of BL_rep_ and BLFU_rep_ measurements (Table 1) were included for all probes. In this model, random measure errors for both time points were taken into account under the assumption that those are independent and normally distributed.

#### Passing-Bablok regression

Passing-Bablok regression [9] extends simple regression models and allows independent measurement errors without making assumptions about error distributions. *PaBALarge* from the R package *mcr* was employed. Since the implementation of Passing-Bablok regression is CPU-intensive for large sample sizes, the number of probes had to be reduced. Therefore, the probe-wise mean expression over all replicates within each batch was used.

#### Linear mixed model

R package *nlme* was used to estimate correction parameters in a linear mixed model. All probes in all BL_rep_ / BLFU_rep_ sample pairs (Table 1) were included for testing. Expression values for BLFU_rep_ measurements were used as dependent, BL_rep_ sample measures as independent variables. Since expression values not only depend on batch membership, but also on the biological condition, it is reasonable to take the sample origin into account. In the replicates, biological differences are reflected by the GHS individual the RNA was isolated from. In the linear mixed model, duplicates / triplicates of samples were assigned to GHS individuals by the random variable.

#### Polynomial regression

Non-linear relationships between expression of BL_rep_ and BLFU_rep_ measurements were modeled by multivariate 3^rd^ grade polynomial regression based on standard R function *lm*. As in the linear mixed models, all probes and all samples were employed in this model. An independent variable representing the GHS individual the RNA was isolated from was used to account for biological variation.

#### Qspline

The *qspline* method was conducted as described in [10] to fit expression of each probe and each sample from BL to the mean distribution of all BLFU measurements. Therefore, the function *normalize*.*qspline* from the R/Bioconductor package *affy* was applied.

#### ComBat

Batch effect correction was performed using the function *ComBat* [11] from R/Bioconductor package *sva* [5]. For this purpose, BL_rep_ measurements were treated as first batch and BLFU_rep_ and FU_rep_ as second batch.

#### ReplicateRUV

The function Naive*ReplicateRUV* from the R/Bioconductor package *RUVnormalize*, here referred to as *ReplicateRUV*, was utilized to remove batch effects. *ReplicateRUV* estimates and removes unwanted variation based on negative control genes and sample replicates. The function requires the parameter *k*, an estimated number of factors causing unwanted variation, and a matrix defining sample membership of replicates. To avoid removal of biological variation, only BL_rep_ and BLFU_rep_ replicates were used. Jacobs et al [17] propose to set k to the number of samples / 4 or to the number of replicates, if the latter is smaller than the former. In this study, the median number of replicates per sample from BL_rep_ and BLFU_rep_ is five, thus *k* = 5. However, also *k* = 2 and *k* = 33 (132 samples / 4) was tested.

In a second step, all batch effect correction methods were repeated after batches were quantile normalized separately.

### Assessment of maintained biological variability

Successful batch effect removal methods were evaluated for their capability to preserve biological variability by four approaches.

1. Clustering of BL_rep_ and BLFU_rep_ measurements from 15 GHS individuals was used to assess biological variability. Clustering was visualized using dendrograms. Samples from one GHS individual falling into the same cluster were considered to be a strong indicator for maintained biological effects. Separation of the dendrogram into different time points of hybridization and scanning was rated as an indicator for preserved batch effects.
2. Principal variation component analysis (PVCA) [23] was used to quantify to which degree technical and biological sources affect overall variation. R/Bioconductor package *pvca* was utilized to estimate source and proportion of variation in two steps according to Chen et al [3]: First, PCA was performed in overall gene expression of all replicates. Principal components (PCs) accounting for 60% of total variation were retained for the next step. Secondly, variant component analysis (VCA) was applied to match each PC to selected sources of variation by linear mixed models. Following sources were selected: i) the time point of RNA measurement to quantify batch effects, ii) time point of RNA extraction for biological differences after 5 years, and iii) the ID of the GHS individual representing biological differences between participants and residuals containing remaining variation.
3. Quantile normalization followed by *ComBat* and quantile normalization plus *ReplicateRUV* were compared for their capability to retain biological variation. The underlying assumption of this step is that a gene expression profile is specific for each subject and that a large set of genes does not change its expression patterns over time. Thus, the overall gene expression of an individual should be very similar to measurements of the same individual 5 years later. In 22 iterations, 50 subjects were drawn without replacement from all 1092 GHS individuals. Overall gene expression at BL and FU was hierarchically clustered based on pairwise Euclidian distances. Subsequently, the number of subjects with BL and FU measurements, clustering together was counted.
4. Association analysis of gene expression to a phenotype was used to assess biological variability in 1092 GHS subjects. Association analyses were performed between body mass index (BMI) and transcriptomes. Regressions were performed in following subsets independently: i) quantile normalized BL data, ii) quantile normalized and *ComBat* corrected BL data, iii) quantile normalized FU data and iv) quantile normalized plus *ComBat* corrected FU data. A linear mixed model was applied with the expression as dependent variable and sex, age and BMI as covariates. According to Schurmann et al. [2], technical variation was taken into account by a random variable. Genome-wide significance level was set to a Benjamini-Hochberg based FDR < 0.05 [24]. Correlation coefficients were calculated between beta estimates / log_10_(p-value) from linear mixed models applied in i) and ii) for BL data and between iii) and iv) for FU data respectively.

## Results

### Strong batch effects are present between baseline and 5-year follow up gene expression levels

**Fig 2:**
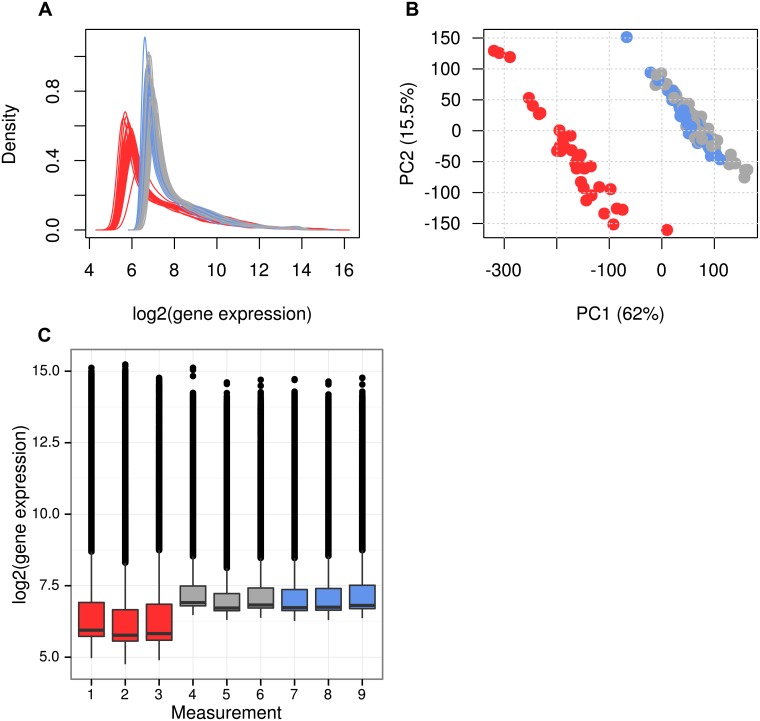
Batch effects between baseline and 5-year follow up samples. Replicates of RNA samples were measured at baseline and follow up. Overall gene expression of those groups were used to generate plots visualizing distributions and variation of transcriptomes. A: Density plots show a clear shift between samples measured at baseline and follow up, with higher expression at 5-year FU measurement. B: The plot of principal component (PC) 1 against PC2 from PC analysis indicate strong batch effects between examination dates. Measurements from the same date (BLFU_rep_ and FU_rep_) are very similar to each other, although RNA was extracted 5 years apart. C: Boxplots of 9 measurements from the same GHS individual grouped by BL_rep_, BLFU_rep_ and FU_rep_. Again, distributions of samples measured within the same batch are very similar to each other. BL_rep_: RNA extracted and measured at BL, BLFU_rep_: RNA extracted at BL and measured at FU, FU_rep_: RNA extracted and measured at FU. Red: Replicates from the group of BL_rep_ measurements, Blue: BLFU_rep_ measurements and dark grey: FU_rep_ measurements.

Expression levels were detected for 20,399 probes in 1092 GHS subjects at baseline (BL) and 5-year follow up (FU). To distinguish batch effects from biological differences between BL and FU, RNA of 15 randomly selected GHS individuals with samples available at both time points and measured in duplicates or triplicates as depicted in Table 1 and Fig 1B and 1C. Overall expression differences between RNA isolated and measured at BL (BL_rep_) and RNA isolated at BL and repeatedly measured at FU (BLFU_rep_) represent batch effects. Differences between BL_rep_ measurements and replicates isolated and measured at FU (FU_rep_) are explained by batch effects and longitudinal gene expression changes (Fig 1B and 1C). Strong differences of overall gene expression distributions for replicates between both time points were observed for the replicates (Fig 2A), and principal component analysis (PCA) clearly separated BL from FU samples (Fig 2B). FU_rep_ samples fell into one cluster with BLFU_rep_, and they were separated from BL_rep_ samples pointing towards strong batch effects. Distributions of overall gene expression can clearly be assigned to batches instead of biological time points as shown for measurements from one subject in Fig 2C. This was observed for all 15 individuals with available BL_rep_, BLFU_rep_ and FU_rep_ measurements (S1 Fig). When differential gene expression analysis between BL_rep_ and BLFU_rep_ was performed, more than 90% of all probes were significantly differentially expressed at a false discovery rate (FDR) < 0.05, showing that this analysis is influenced by batch effects. Potential sources of technical variation were i) dilution, labeling and hybridization of RNA at different study sites by different personnel, ii) distinct versions of the Illumina HT12 microarray and iii) the use of different Illumina microarray scanners (Fig 1A).

### *ComBat* and *ReplicateRUV* efficiently reduced batch effects in the study subset of replicates

**Fig 3:**
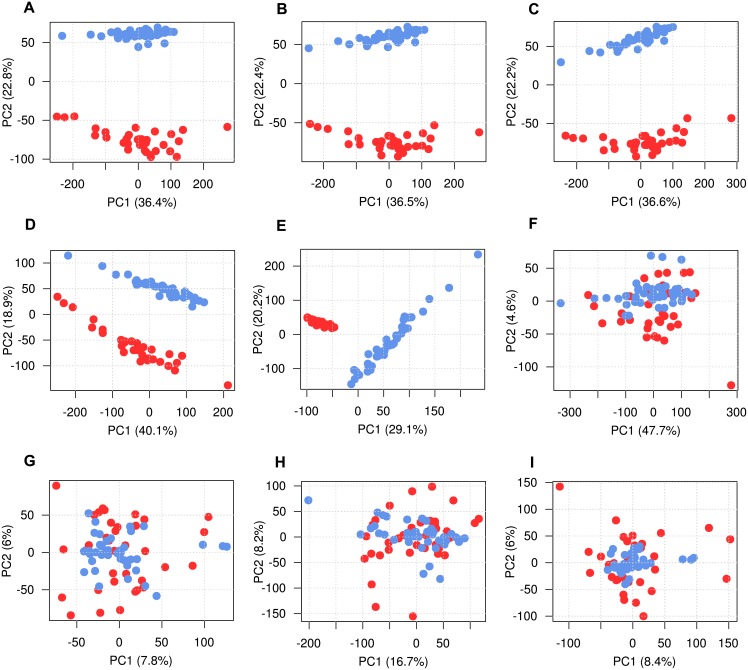
Comparison of different batch effect removal approaches. Replicates of RNA samples extracted at BL were hybridized on Illumina HT12 microarrays at both examination dates. Overall gene expression rescaled by seven different approaches. Components of variance are visualized as PCA plots. Replicate samples extracted and measured at baseline (BL_rep_) are marked red and repeated measures at 5-year follow up (BLFU_rep_) in blue. Correction based on A: Deming regression, B: Passing-Bablok regression, C: linear mixed models, D: 3^rd^ order polynomial regression and E: *qspline* [10] was not capable to remove batch effects from gene expression data. The PCA plots show clusters between replicates extracted, processed and hybridized at both time points. F: After applying *ComBat*, G: quantile normalization followed by *ComBat*, H: *ReplicateRUV* and I: quantile normalization plus *ReplicateRUV*, no clustering of samples was observed indicating successful removal of batch effects.

In order to find the best approach for batch effect removal, 7 methods to adjust for batch effects were compared: i) Deming regression, ii) Passing-Bablok regression, iii) linear mixed models, iv) 3^rd^ order polynomial regression, v) *qspline*, vi) *ComBat and vii) ReplicateRUV*. Each method was first tested on raw log_2_-transformed data and, in a second step, after quantile normalization of batches. For the first four methods, differences in gene expression between BL_rep_ and BLFU_rep_ measurements were estimated by the respective regression model. Effect estimates were then used to correct batch effects in BL_rep_ samples by rescaling gene expression levels of all probes. For methods v),vi) and vii) R/Bioconductor packages *affy*, *sva* [5] and *RUVnormalize* [17] were applied to all BL_rep_ and BLFU_rep_ samples. Variation attributable to batch effects before and after batch adjustment were identified using plots of principal component analysis (PCA) and principal variation component analysis (PVCA). PCA plots for all methods are depicted in Fig 3. The number of differentially expressed genes and the estimated proportion of variance explained by batch and potential biological differences for each method is summarized in Table 2.

**Table 2:** Comparison of batch effect removal methods.

	No. differentially expressed genes between replicates measured at both time points		PVCA (%)			
	Benjamini-Hochberg^a^^)^	Bonferroni^b^^)^	Batch	Time point	Individuals	Residuals
Uncorrected data	18,526 (90.8%)	15,631 (76.6%)	35.7	3.4	37.6	23.2
Deming regression	14,939 (73.2%)	8,137 (39.9%)	35.7	3.4	37.6	23.2
Passing-Bablok regression	14,257 (69.9%)	7,935 (38.9%)	35.7	3.4	37.6	23.2
Linear mixed model	14,147 (69.4%)	7,933 (38.9%)	35.9	3.4	37.7	22.9
Polynomial regression	14,558 (71.4%)	7,936 (38.9%)	34.0	3.6	38.0	24.4
Qspline	14,771 (72.4%)	8,700 (42.6%)	34.4	3.5	37.7	24.4
*ComBat*	0 (0%)	0 (0%)	4.6	2.7	55.1	37.6
QN + *ComBat*	0 (0%)	0 (0%)	4.2	2.5	56.7	36.6
*ReplicateRUV*	0 (0%)	0 (0%)	0.3	9.8	59.7	30.2
QN+*ReplicateRUV*	0 (0%)	0 (0%)	0.1	8.1	65.0	26.8

Batch effect removal was evaluated in terms of differential expression between replicates and principal variance component analysis (PVCA) after correcting overall gene expression. Differential expression was calculated between 35 RNA replicates isolated and measured at baseline and 43 RNA replicates isolated at baseline and hybridized at 5-year follow up. Since both groups were isolated from the same monocytic cells and differ only by the measurement time point, observed differences can be assigned to batch effects. In uncorrected data, more than 90% of all probes were significantly differentially expressed. *ComBat* and *ReplicateRUV* eliminated differential expression between batches. None of the other approaches notably reduced the number of differentially expressed genes. For PVCA, additionally 54 RNA replicates isolated and hybridized at follow up were included in the analysis. PVCA was applied after batch effect correction to estimate the proportion of variance on overall gene expression variation explained by the batches (Batch), potential biological differences after 5 years (Time point), potential biological differences between 15 individuals the RNA was isolated from (Individual) and remaining variation (Residual). The estimated explained variance attributable to batches was largely reduced after applying *ComBat*. *ReplicateRUV* almost fully removed batch effects. Quantile normalization (QN) prior to *ComBat* or *ReplicateRUV* slightly improved batch effect removal. The other approaches did not reduce batch effects.

^a:^ significance level defined as Benjamini-Hochberg corrected FDR < 0.05;

^b:^ significance level defined as Bonferroni corrected p-value < 0.05.

**Fig 4:**
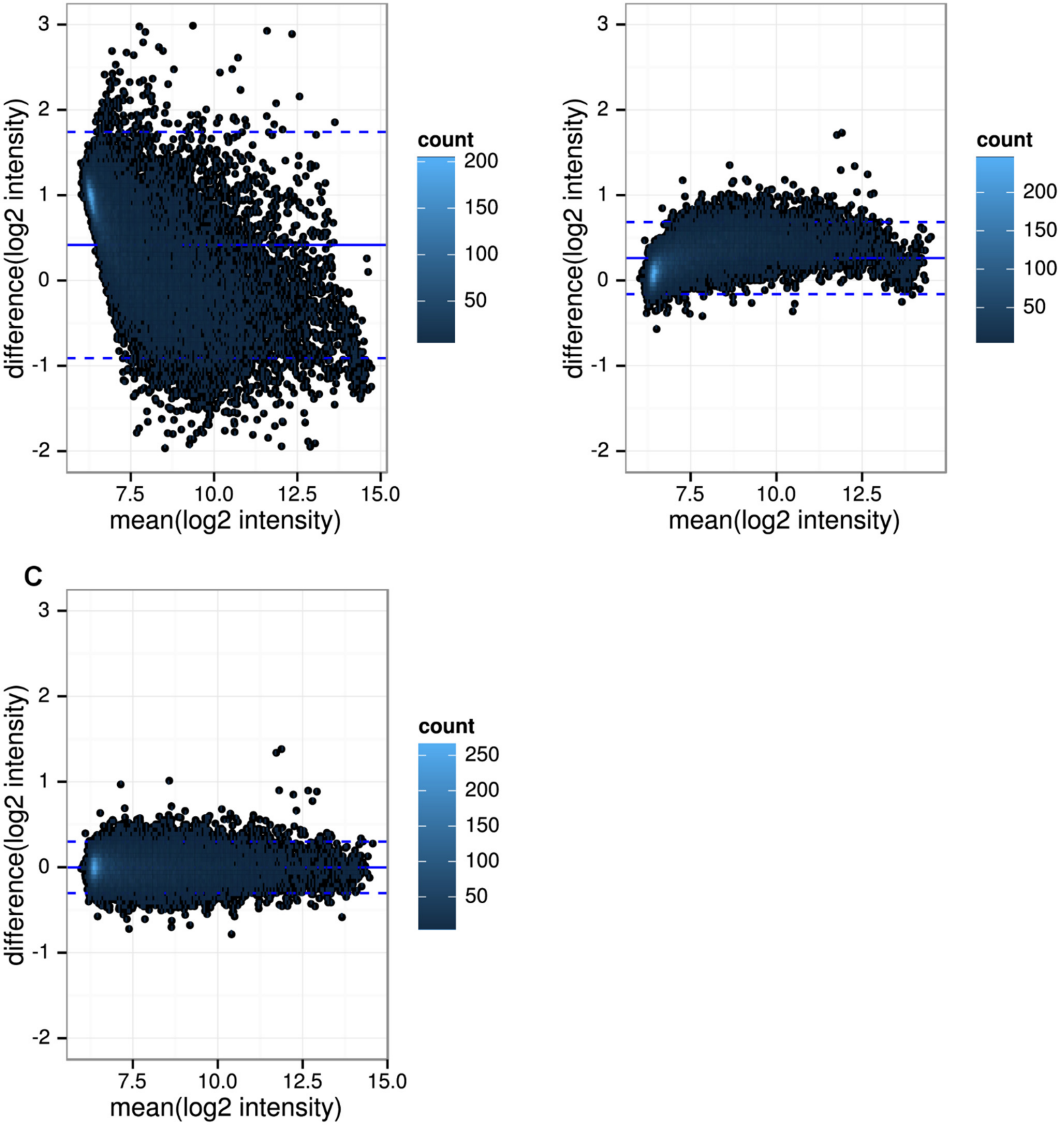
Comparison of gene expression data between baseline and 5-year follow up. Bland-Altman plots were produced using microarray data from one GHS individual to evaluate agreement of repeated measures before and after batch effect removal. Expression differences between technical replicates hybridized at baseline and 5-year follow up were therefore plotted against the mean expression of both time points for each probe. Each dot represents one probe and dense clusters of probes are marked blue. The majority of probes had low expression values. A: In uncorrected data, large expression differences between baseline and follow up data could be observed. Those differences were strongly dependent on the mean expression. B: After applying *ComBat*, differences were largely reduced, but were increased for probes with high expression. C: When quantile normalization was performed separately in each batch followed by *ComBat*, the best results were obtained in terms of agreement between repeated measures.

Gene expression correction based on estimates from linear models (Fig 3A–3C) was not capable to remove batch effects as indicated by clusters between BL_rep_ and BLFU_rep_ samples. Quantile normalization, performed separately in both batches prior to batch effect correction, did not improve the methods (S2A–S2C Fig). Overall variance explained by batches was not remarkably reduced compared to uncorrected data and was still around 50% (Table 2). Expression differences between uncorrected BL replicates measured at BL and FU were plotted against mean expression from both batches using Bland-Altman plots for one GHS participant [25]. As shown in Fig 4A (and S3 Fig), gene expression differences depended on measured gene expression levels, while they were highest for low gene expression values. A non-linear relation was observed between gene expression levels at baseline and those repeatedly measured at follow up. However, neither third-order polynomial regression nor the non-linear method *qspline* led to a substantial reduction of technical variation (Fig 3D and 3E and S2D Fig). The proportion of genome-wide significantly differentially expressed Illumina probes between batches remained high for linear and non-linear model based correction, ranging from 69.4% (linear mixed models) to 73.2% (Deming regression).

**Fig 5:**
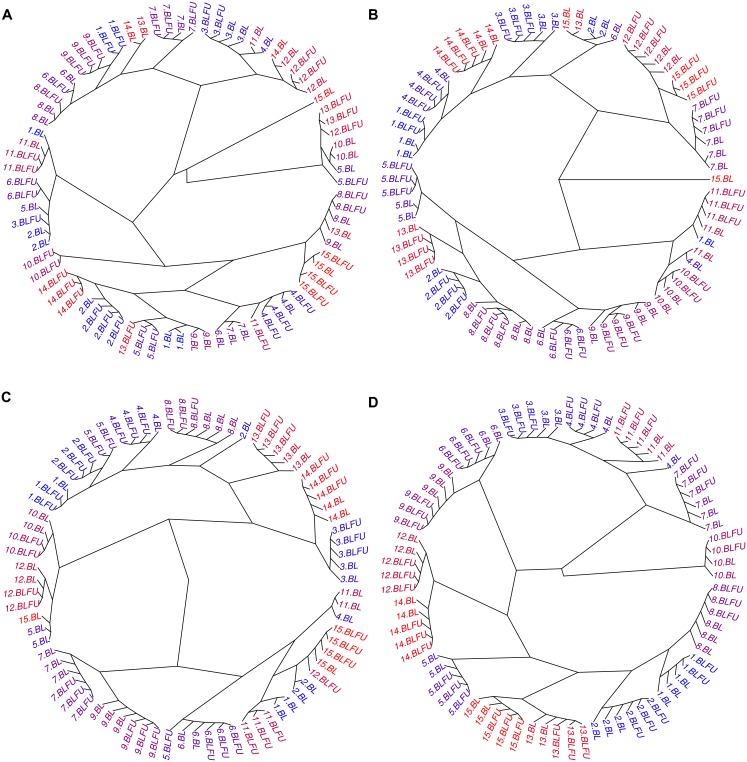
Clustering of sample replicates after batch effect correction. RNA samples extracted at BL and measured at BL (BL_rep_) or FU (BLFU_rep_) were clustered based on pairwise distance of overall gene expression. Each GHS individual is represented with an ID between 1 and 15. Batch membership is indicated by the labels BL for BL_rep_ and BLFU for BLFU_rep_. RNA used for hybridization and scanning was utilized from the same stock at both time points. As a consequence, overall gene expression for one individual should be very similar between technical replicates. Before batch effect correction, samples fell into two clusters representing batches. A: After applying *ComBat* clustering improved. B: Quantile normalization plus *ComBat* led to clusters, which mainly discriminate between individuals, indicating retained biological effects. C: *ReplicateRUV* led to comparable results and D: quantile normalization plus *ReplicateRUV* led to almost perfect classification.

Next, gene expression levels of BL and FU samples were jointly corrected using *ComBat* [11]. As illustrated in the PCA plot, no clustering of sample replicates into batches was observed (Fig 3F). None of the probes showed differences in gene expression levels between BL_rep_ and BLFU_rep_ samples at an FDR of 0.05 (Table 2), indicating a great improvement as compared to the more than 90% differentially expressed genes from the uncorrected data sets. The estimated proportion of batch effects on total variation was reduced to 4.6% after *ComBat*, and the explained variance by differences between individuals was 55.1% (Table 2). Quantile normalization prior to *ComBat* led to a slightly improved batch effect removal (4.2%). *ReplicateRUV* almost removed batch effects with an estimated explained variation of 0.3% by batches and 59.7% by differences between individuals. Previous quantile normalization led to decreased technical variation (0.1%) making biological variation more prominent (65%). In the next step, removal of technical variation and retention of biological effects were investigated by hierarchical clustering based on pairwise Euclidian distances of all BL_rep_ and BLFU_rep_ measurements from the 15 GHS subjects. Observed gene expression between BL_rep_ and BLFU_rep_ should only differ by batch effects. Thus, a successful batch effect removal should ideally lead to 15 clusters, one for each individual with mixed BL_rep_ and BLFU_rep_ measures. Clustering results were visualized as dendrograms in Fig 5. After *ComBat*, replicates from 9 individuals were partly clustered correctly, meaning that some (but not all) BL_rep_ and BLFU_rep_ measures of one individual fell into the same cluster (Fig 5A). Combining *ComBat* with quantile normalization greatly improved clustering, leading to 8 correct and 6 partly correct classifications (Fig 5B). *ReplicateRUV* performed comparably with 8 proper and 4 partly correct clusters (Fig 5C). Quantile normalization followed by *ReplicateRUV* resulted in an almost perfect classification, with just one replicate classified incorrectly. In summary, quantile normalization plus *ComBat* or *ReplicateRUV* substantially reduced batch effects in the dataset containing replicates of 15 GHS individuals.

### *ComBat* outperformed *ReplicateRUV*, when applied in the whole study dataset

**Fig 6:**
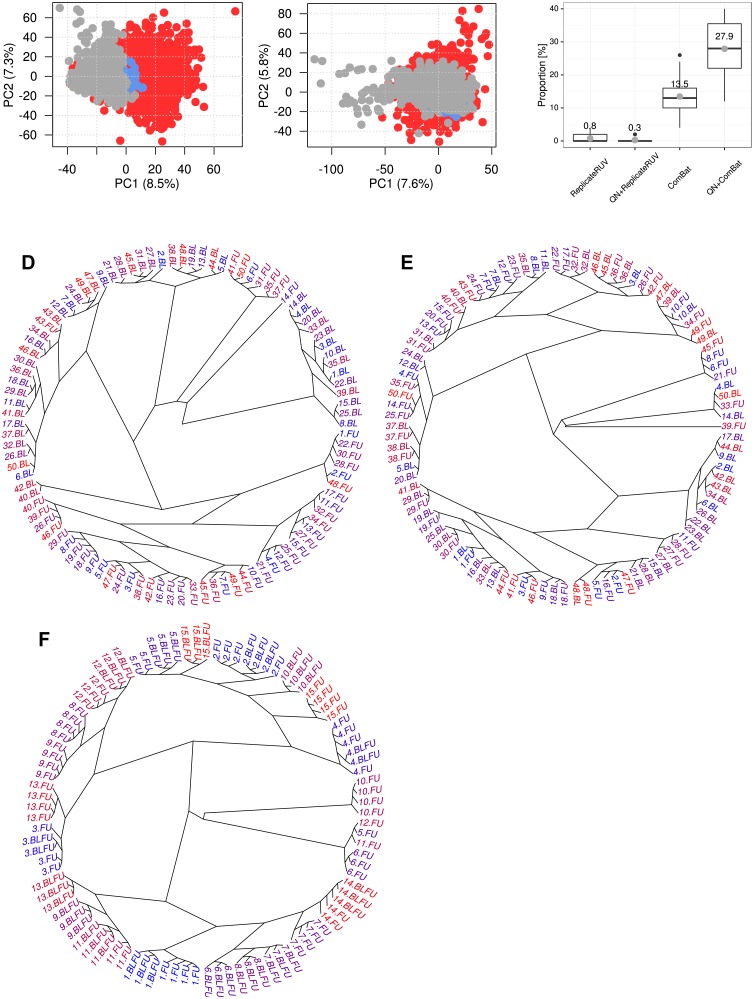
Comparison of batch effect removal by *ReplicateRUV* and *ComBat* in the full dataset. Overall gene expression was corrected for batch effects by either *ReplicateRUV* or *ComBat*. A, B: Components of variance are visualized as PCA plots. Samples extracted and measured at baseline (BL) are marked red, repeated measures at 5-year follow up (BLFU_rep_) in blue and follow-up (FU) samples in grey. Batch correction based on A: quantile normalization plus *ReplicateRUV* resulted in clusters indicating remaining batch effects, while B: quantile normalization plus *ComBat* removed those effects. C: 50 subjects with BL and FU gene expression data available were drawn from 1092 individuals in 22 iterations. Gene expression was hierarchically clustered and the number of subjects with BL and FU falling into direct proximity was counted. On the y-axis, the proportion of correctly clustered pairs is shown for different batch effect removal approaches. Quantile normalization plus *ComBat* led to the highest proportion of pairs indicating maintained intra-individual similarity between time points. D: Example dendrogram from hierarchical clustering after quantile normalization plus *ReplicateRUV*. Each individual is represented with an ID between 1 and 50. The labels BL and FU represent time points. One subject was identified with “BL” and “FU” (BL: Baseline, FU: 5y follow-up) clustered in direct proximity (40.BL, 40.FU). E: Clustering based on quantile normalized and *ComBat* corrected data led to 16 individuals with BL and FU in the same cluster. F: Hierarchical clustering of quantile normalized data for 15 subjects with BLFU_rep_ and FU_rep_ measurements. Here, for 7 individuals (46.7%) all measurements fell into the same cluster.

The aforementioned analyses were performed in a subset of 15 individuals with repeated measures of up to 6 replicates per sample. Next, quantile normalization followed by *ComBat* or *ReplicateRUV* was applied in the entire dataset of the study. Principal component analyses of corrected gene expression showed more prominent clusters between BL and FU samples for *ReplicateRUV* (Fig 6A) compared to *ComBat* (Fig 6B). Clusters between batches were also observed for *ReplicateRUV* when using different values for the parameter *k* (S4 Fig). In *ReplicateRUV* corrected data, 17,974 (88.1%) out of 20,399 probes were differentially expressed between BL and FU at a FDR ≤ 0.05, indicating that batch effects were still present. In contrast, *ComBat* corrected data did not show any significant differential expression between time points. To study, whether biological variation was preserved after batch effect correction, 22 subsets, each containing 50 individuals, were built and used for hierarchical clustering. The underlying assumption was that intra-individual biological variation between time points is on average lower than inter-individual differences of gene expression profiles. To evaluate this assumption, BLFU_rep_ and FU_rep_ measurements were clustered (Fig 6F). Both groups were processed and scanned at FU. Thus, variation between BLFU_rep_ and FU_rep_ for one individual should reflect biological differences after 5 years. For 7 out of 15 individuals (46.7%), all replicates from both time points fell into the same cluster. This indicated that the assumption holds for a substantial proportion of individuals. An exemplifying dendrogram for quantile normalized plus *ReplicateRUV* corrected data is shown in Fig 6D. Only one individual (43.BL and 43.FU) was found in direct proximity for this subset. Fig 6E shows an example for quantile normalized and *ComBat* corrected data. Here, 16 out of 50 (32%) individuals were found with BL and FU within one cluster. The proportions of intra-individual pairs per 50 samples are summarized in Fig 6C. The mean proportion was 0.8% after *ReplicateRUV* and 0.3% after quantile normalization plus *ReplicateRUV*. Batch effect correction by *ComBat* increased the proportion of pairs to 13.5%. The best results were achieved after quantile normalization and *ComBat* with a mean proportion of 27.9%. Similar results can be observed, when comparing Euclidian distances without subsequent clustering (S5 Fig). In addition, Bland-Altman plots showed that the latter approach led to an increased reproducibility of repeated measures when compared to *ComBat* alone (Fig 4B and 4C). Taken together, quantile normalization plus *ComBat* efficiently removed batch effect and retained intra-individual variation.

### Quantile normalization plus *ComBat* maintained biological variability

Approaches to remove batch effects should not eliminate biological variation. Hence, batch effect removal was evaluated for their capability to preserve biological variability by comparing summary statistics of association analysis between body mass index (BMI) and gene expression in two different settings.

**Fig 7:**
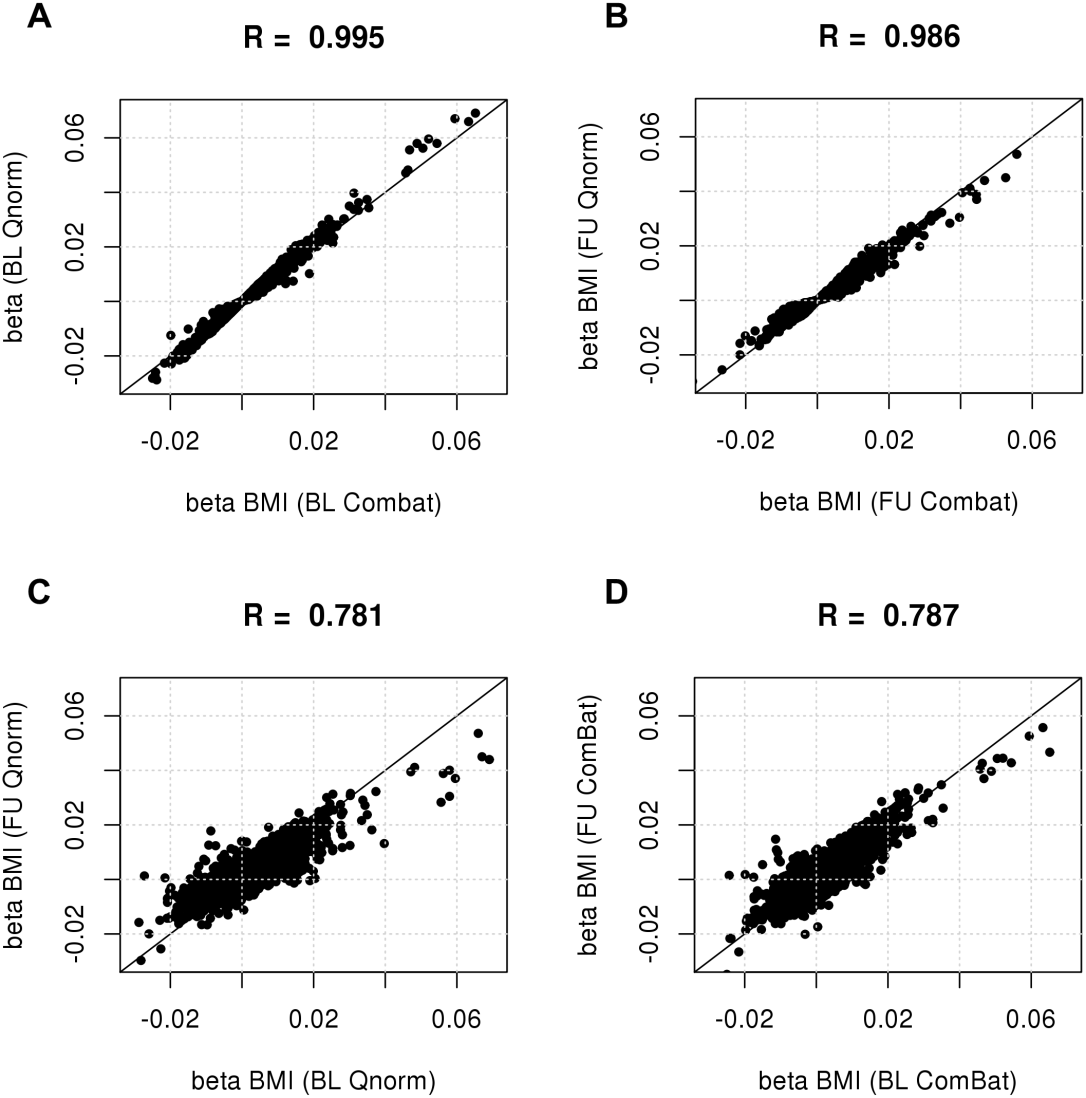
Maintenance of biological variation after quantile normalization and *ComBat*. To assess whether biological sources of variability were maintained after batch effect removal, associations between each probe and body mass index (BMI) were calculated using linear mixed models within each batch containing 1092 samples before and after applying *ComBat*. A, B: For each probe, we plotted the effect of BMI on expression in ComBat corrected data on the x-axis and the quantile-normalized but uncorrected on the y-axis. BMI beta estimates were highly correlated between corrected and uncorrected datasets in A: BL samples (R = 0.995) and B: FU samples (R = 0.986). C, D: The effect of BMI on gene expression in BL samples was plotted against the effect observed in FU samples after C: quantile normalization and D: quantile normalization followed by *ComBat*. The correlation between BMI effect estimates at BL and FU was slightly higher for *ComBat* corrected data (r = 0.787) compared data, which was only quantile normalized (r = 0.781).

First, we investigated whether *ComBat* reduced biological variation within one batch, i.e. within BL or FU data. Associations between BMI and each probe were calculated in quantile-normalized data individually before and after batch effect removal. Within the BL data, 1498 probes were significantly associated with BMI at a FDR<0.05 and after application of *ComBat*, indicating that rescaling of gene expression during batch effect removal did not affect biological variation. The same result was observed in FU samples with 2281 significant associations before and after batch correction. Effect estimates of BMI probe associations were highly correlated between datasets in BL (r = 0.995) and FU (r = 0.986) (Fig 7A and 7B). Differing effect sizes between datasets can be explained by change in the overall expression levels and variance after *ComBat*. All probes with deviating effect estimates showed differences in standard errors. P-values from BMI association tests were almost identical between quantile normalized and *ComBat* corrected data (S6 Fig).

In a second step, summary statistics of BMI associations were compared between the two time points. Since all subjects were five years older at the time of the follow up, a perfect correlation between BL and FU was not expected. However, they should be comparable. BMI effect estimates of quantile normalized BL data were correlated with those from quantile normalized FU data (r = 0.781) (Fig 7C). Larger effect sizes were lower in FU compared to BL data. The correlation between BMI effect estimates from BL and FU after quantile normalization and *ComBat* was slightly higher (r = 0.787) (Fig 7D) when compared to the correlation in quantile normalized data. Differences between large effect estimates from BL and FU data became less prominent.

Taken together with the results from hierarchical clustering, these findings clearly show maintained biological variability of overall gene expression after batch effect removal through quantile normalization followed by *ComBat*.

## Discussion

The combination of quantile normalization and *ComBat* in large-scale, longitudinal gene expression data is the best approach for removal of batch effects in our study dataset. Observed batch effects between BL and FU replicated samples were strong. Probes with low gene expression levels were > 2-fold higher in FU compared to BL. In contrast, expression levels were comparable for medium to high signal intensities. Batch effects were largely reduced from an estimated proportion of 35.7% on overall variation to 4.2%. When focusing on the dataset of sample replicates, *ReplicateRUV* outperformed *ComBat* in terms of batch effect removal and retention of biological variation. Technical variation between batches was almost fully removed after quantile normalization and *ReplicateRUV*, leaving 0.1% estimated explained variation by batch effects. Sample replicates repeatedly measured at both time points (BL and FU), fell into virtually perfect clusters indicating maintained biological variation. However, when batch effect removal was applied on the full dataset of 1092 GHS subjects, quantile normalization plus *ComBat* performed better compared to quantile normalization followed by *ReplicateRUV* or *ReplicateRUV* alone. Different numbers of *ReplicateRUV* parameter k, which gives an estimate of the number of unwanted factors, were tested. The estimates did not substantially improve batch effect removal, leaving the relatively low proportion of replicates compared to the large study cohort as potential cause for these divergent results.

After quantile normalization plus *ComBat*, biological variation was preserved as shown by repeated measures of RNA replicates, maintained intra-individual similarity and by BMI association analyses in all 1092 GHS participants with expression data available at both time points. All other approaches, i.e., Deming regression, Passing-Bablok regression, linear mixed models and non-linear approaches did not adequately reduce the batch effects. Thus, quantile normalization followed by *ComBat* was the only approach tested which successfully removed batch effects.

Quantile normalization followed by *ComBat* performed better than *ComBat* alone. As indicated by hierarchical clustering and analysis of inter- and intra-individual distances, quantile normalization prior to batch effect removal led to improved retention of biological signals. In addition, quantile normalization, performed separately in batches, led to a slightly improved reduction of technical variation. Briefly, *ComBat* works in three steps: i) data standardization, ii) empirical estimation of prior distribution hyperparameters from standardized data and subsequent estimation of batch effect parameters, which are iii) used to correct batches. The first step aims to reduce biases, when estimating hyperparameters. Distributions of overall gene expression in follow-up samples were narrower and more skewed towards zero, had lower interquartile ranges and altogether higher values compared to the other batch. Quantile normalization reduced variances within each batch and maintained ranking of genes. Thus, a possible explanation for the observations is that prior quantile normalization helped to facilitate bias reduction during batch effect parameter estimation by *ComBat* and therefore led to an improved batch effect removal.

The successful application of *ComBat* reported in this study is the first report on performance of *ComBat* in large-scale longitudinal gene expression data and confirms data from the current literature. Kitchen et al. [4] used repeated hybridizations of human reference RNA replicates in 18 chips spread over 5 batches to evaluate transcriptome variation within and across batches. Both, intra- and inter-batch correlation between replicates greatly increased after quantile normalization within each batch plus *ComBat*. Chen et al. investigated 6 methods for batch effect removal using two microarray datasets from brain RNA samples and two simulated datasets [3]. *ComBat* outperformed the other 5 methods by most metrics. Quantile normalization plus *ComBat* was also recommended for batch effect removal in Illumina methylation data [26]. *ComBat* was capable to combine different datasets from GEO (ncbi.nlm.nih.gov/geo) as shown by Chmielewski et al. for differential expression analysis of atherosclerotic plaques [14]. Cross-platform integration of microarray data from Illumina and Affymetrix was reported to produce meaningful results when applying *ComBat* [27].

A limitation of this work is that the sources for the strong batch effects are confounded by variation in time point of measurement and changes in the microarray version. However, the technology of Illumina HT12 BeadChip arrays, used in this study remained stable over time and less than 20% of all designed probes differ between versions 3 and 4. It can thus be assumed that shifts in overall gene expression levels represented by light signal intensities can primarily be attributed to scanning using *BeadArray Reader* at BL and *iScan* at FU, respectively (Fig 2). *ComBat* has also been shown to be a valuable approach for batch effect removal from oligonucleotide-based Affymetrix chips [28]. However, in this study, RNA hybridization was performed on Illumina HT12 BeadChip arrays only and results cannot be generalized to other platforms.

In summary, quantile normalization followed by *ComBat* is the best approach for removing batch effects when applied to large-scale longitudinal gene expression data. All other approaches investigated in this work failed. Batch effects were largely removed by *ComBat* as indicated by repeated measures of RNA replicates. Evaluation based on biological replicates showed that biological variation after *ComBat* was maintained. BMI association analyses performed separately before and after applying *ComBat* additionally pinpointed towards maintained biological variation, which is essential for future association analyses between changes in gene expression and clinical phenotypes over time in GHS.

## Supporting Information

S1 Fig Batch effects between baseline and 5-year follow up samples. Boxplots of samples summarized for each of the 15 GHS individuals measured in distinct batches (BL and BLFU) and with RNA isolated and measured 5 years later. Distributions clearly indicate that batch effects outweigh biological effects.
(PDF)

S2 Fig Comparison of different batch effect removal approaches. Replicates of RNA samples extracted at BL were hybridized on Illumina HT12 microarrays at both examination dates. Overall gene expression was quantile normalized batch-wise and rescaled between batches by four different approaches. Components of variance are visualized as PCA plots. Replicate samples extracted and measured at baseline (BL_rep_) are marked red and repeated measures at 5-year follow up (BLFU_rep_) in blue. The PCA plots show clusters between replicates extracted, processed and hybridized at both time points for correction based on A: Deming regression, B: Passing-Bablok regression, C: linear mixed models, D: 3^rd^ order polynomial regression.
(TIF)

S3 Fig Direct comparison of expression between batches. Bland-Altman plots were produced using microarray data from one GHS individual to evaluate agreement of repeated measures before batch effect removal. Expression differences between technical replicates hybridized at baseline and 5-year follow up were therefore plotted against the mean expression of both time points for each probe. Each dot represents one probe and dense clusters of probes are marked blue. The majority of probes had low expression values. In uncorrected data, large expression differences between baseline and follow up data could be observed. Those differences were strongly dependent on the mean expression.
(PDF)

S4 Fig Comparison of different parameters for *ReplicateRUV*. Different values for the parameter k, for the specification of an estimated number of unwanted factors of variation, were tested. Components of variance are visualized as PCA plots. Samples extracted and measured at baseline (BL) are marked red, repeated measures at 5-year follow up (BLFU_rep_) in blue and follow-up (FU) samples in grey. Batch correction based on *ReplicateRUV* with A: k = 2, B: k = 5 and C: k = 33 was performed.
(TIF)

S5 Fig Euclidean distances between samples before / after batch effect correction. Intra-individual variation between time points was calculated by pairwise Euclidean distances between BL and FU for each individual. Inter-individual variation is specified by the mean Euclidean distance between one individual and all other individuals. A-C: replicate data set. A: Quantile normalized data from BLFU_rep_ and FU_rep_ samples—measured within one batch—mainly reflect biological differences and results are thus used as a reference. In contrast, observed differences between BL_rep_ and FU_rep_ include batch effects and biological variation between time points. A batch effect removal strategy that retains biological variation should therefore result in distributions comparable to A. Mean Euclidean distances are shown for batch effect removal by B: quantile normalization (QN) plus *ComBat* and C: QN followed by *ReplicateRUV*. The comparison of batch effect removal in the entire dataset by D: QN plus *ComBat* and E: QN plus *ReplicateRUV* indicates that QN followed by *ComBat* achieved the best results.
(TIF)

S6 Fig Maintenance of biological variation after quantile normalization and *ComBat*. To assess whether biological sources of variability were maintained after batch effect removal, associations between each probe and body mass index (BMI) were calculated using linear mixed models within each batch containing 1092 samples before and after applying *ComBat*. A, B: For each probe, we plotted the p-values from *ComBat* corrected data on the x-axis and the quantile-normalized but uncorrected on the y-axis. BMI p-values were almost identical between corrected and uncorrected datasets in A: BL samples and B: FU samples. C.D: The BMI p-values from BL samples was plotted against the p-values observed in FU samples after C: quantile normalization and D: quantile normalization followed by *ComBat*.
(TIF)

S1 Material Equations used for batch effect removal. (PDF)
