# Supplementary material for: Removing Batch Effects from Longitudinal Gene Expression - Quantile Normalization Plus ComBat as Best Approach for Microarray Transcriptome Data
Source: PLoS One. 2016 Jun 7;11(6):e0156594. doi: 10.1371/journal.pone.0156594 (PMC4896498; doi:10.1371/journal.pone.0156594)
Supplement: S1 Fig — Boxplots of samples summarized for each of the 15 GHS individuals measured in distinct batches (BL and BLFU) and with RNA isolated and measured 5 years later. Distributions clearly indicate that batch effects outweigh biological effects. (PDF) [file pone.0156594.s001.pdf]

S1 Fig. Batch effects between baseline and 5-year follow up samples.

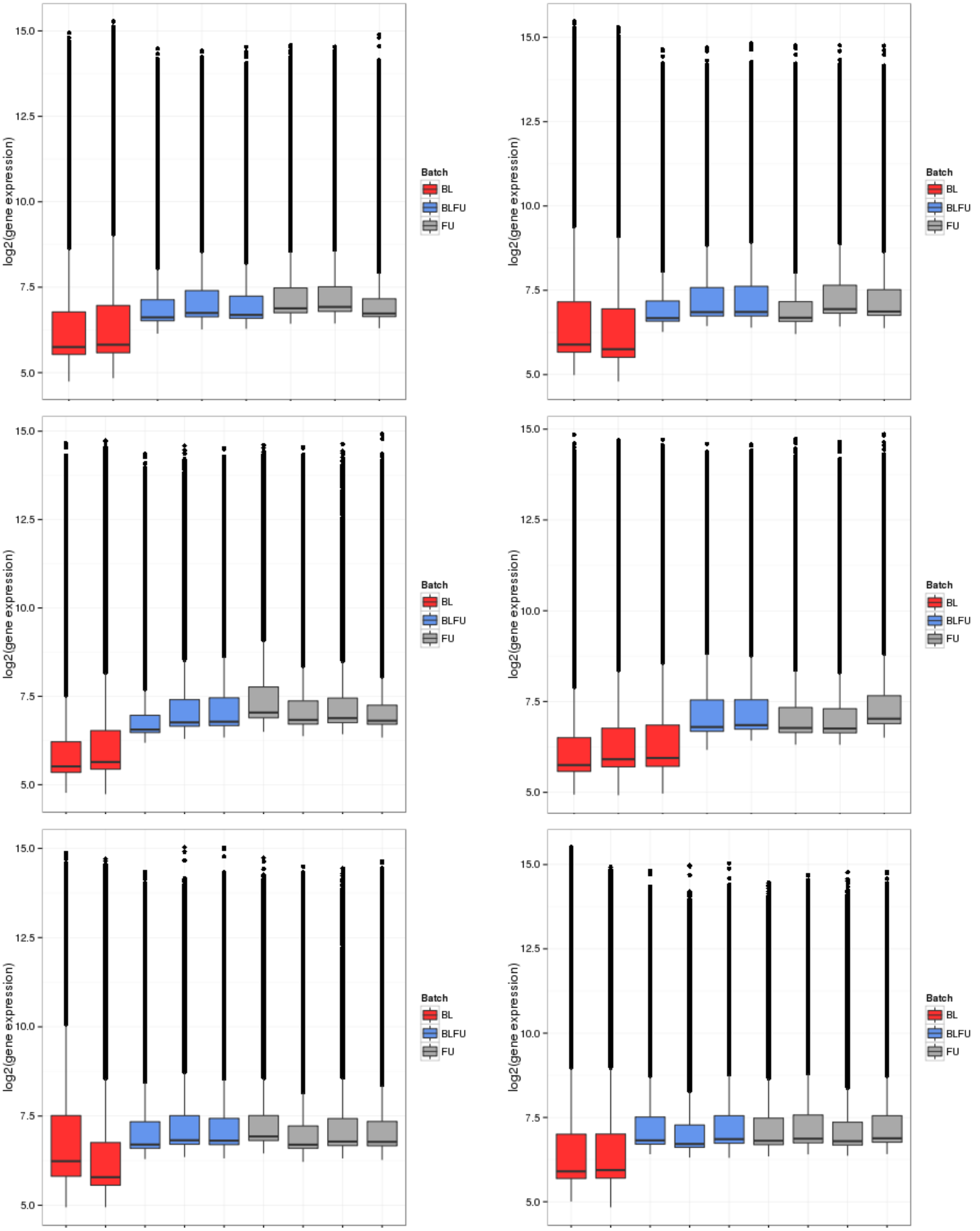

S1 Fig. Batch effects between baseline and 5-year follow up samples.

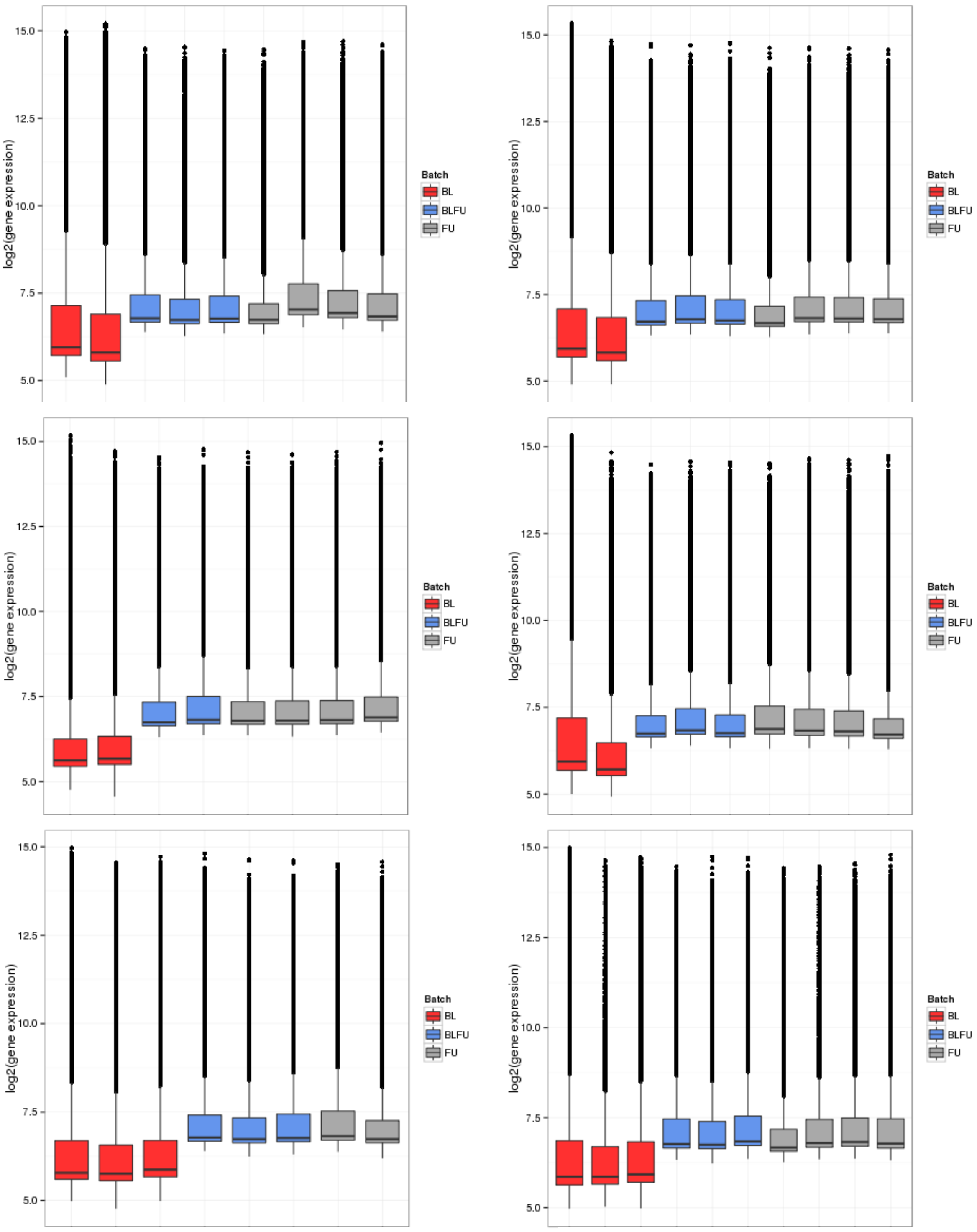

S1 Fig. Batch effects between baseline and 5-year follow up samples.

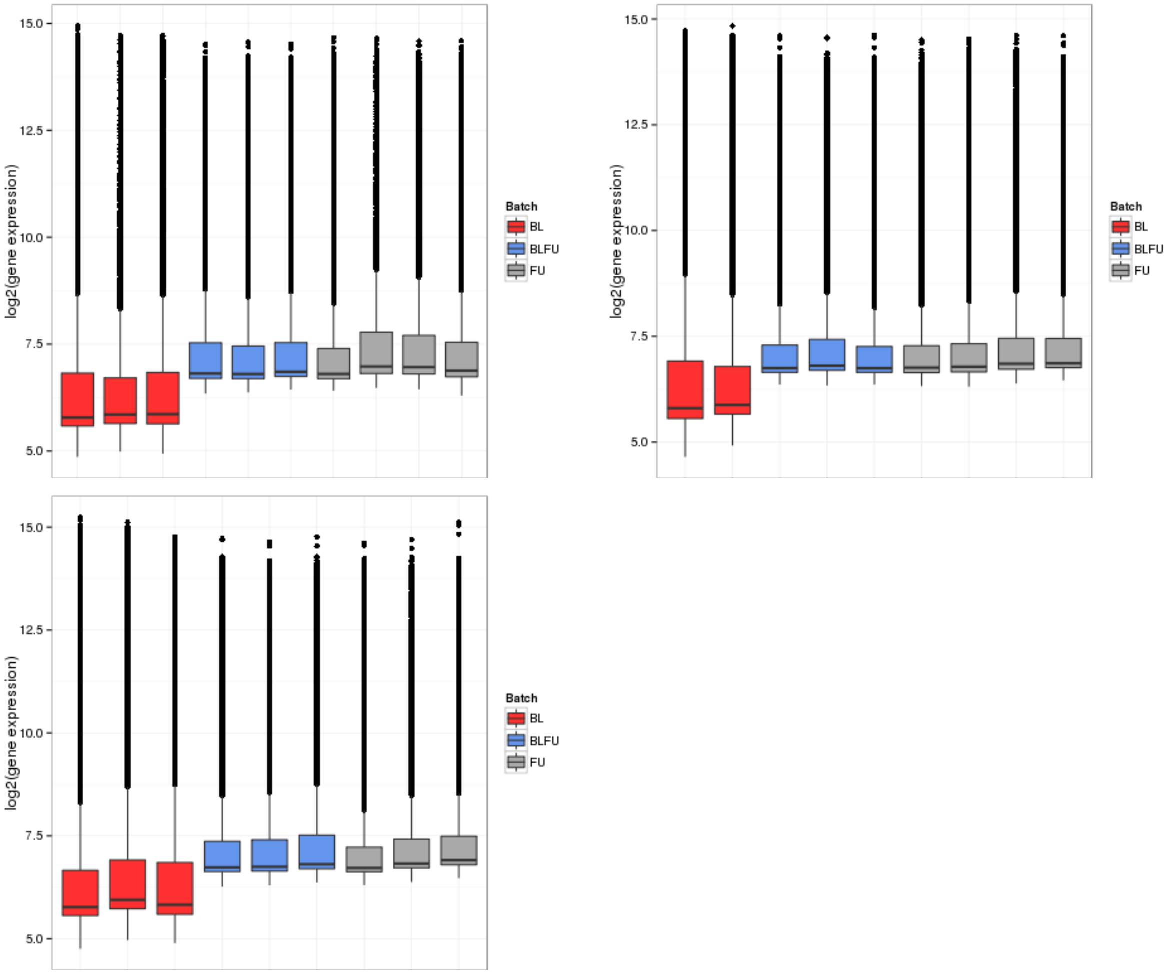

Batch effects between baseline and 5-year follow up samples. Boxplots of samples summarized for each of the 15 GHS individuals measured in distinct batches (BL and BLFU) and with RNA extracted and measured 5 years later. Distributions clearly indicate that batch effects outweigh biological effects.
