# Supplementary figures and images for: Removing Batch Effects from Longitudinal Gene Expression - Quantile Normalization Plus ComBat as Best Approach for Microarray Transcriptome Data
Source: PLoS One. 2016 Jun 7;11(6):e0156594. doi: 10.1371/journal.pone.0156594 (PMC4896498; doi:10.1371/journal.pone.0156594)

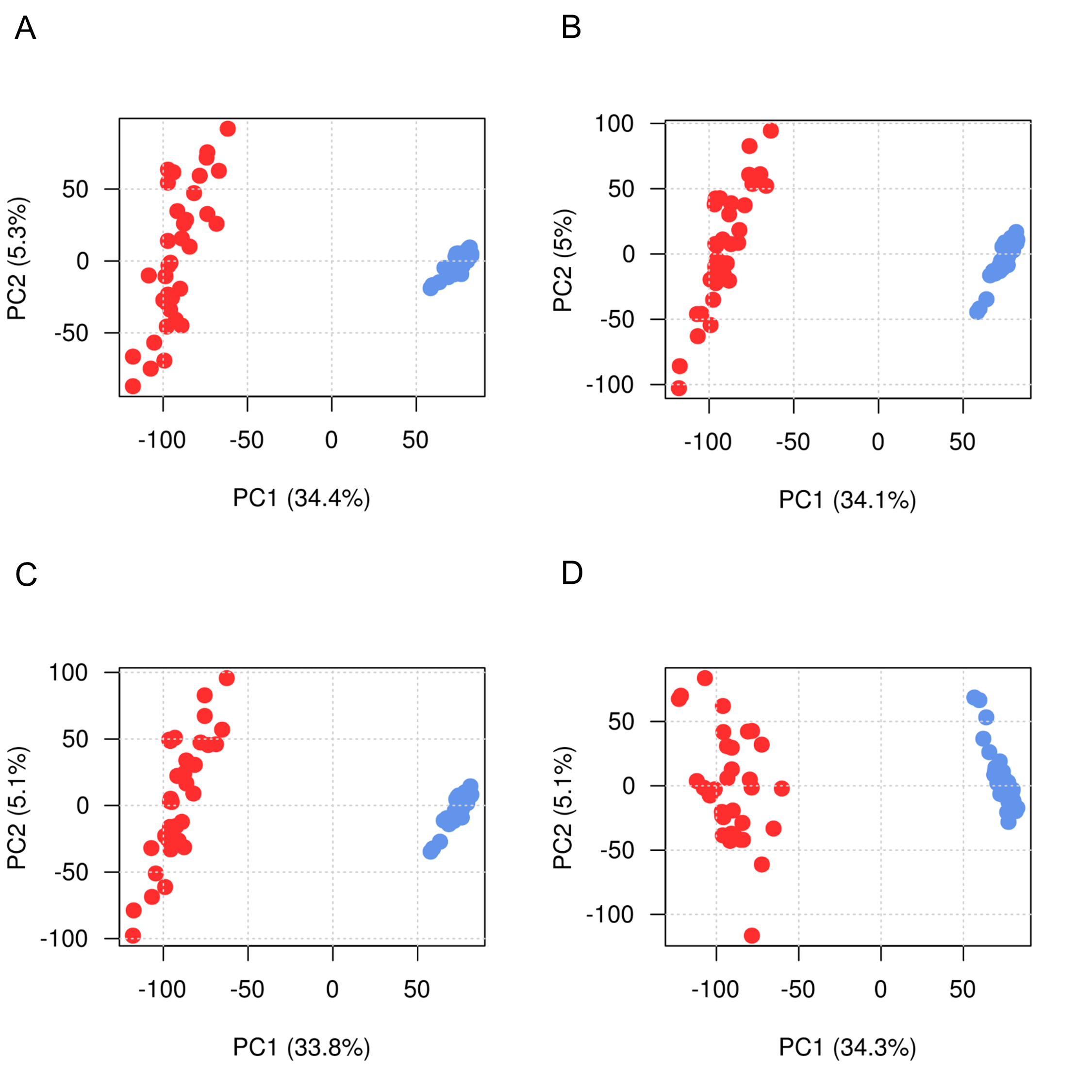

Supplement: S2 Fig — Replicates of RNA samples extracted at BL were hybridized on Illumina HT12 microarrays at both examination dates. Overall gene expression was quantile normalized batch-wise and rescaled between batches by four different approaches. Components of variance are visualized as PCA plots. Replicate samples extracted and measured at baseline (BLrep) are marked red and repeated measures at 5-year follow up (BLFUrep) in blue. The PCA plots show clusters between replicates extracted, processed and hybridized at both time points for correction based on A: Deming regression, B: Passing-Bablok regression, C: linear mixed models, D: 3rd order polynomial regression. (TIF) [file pone.0156594.s002.tif]

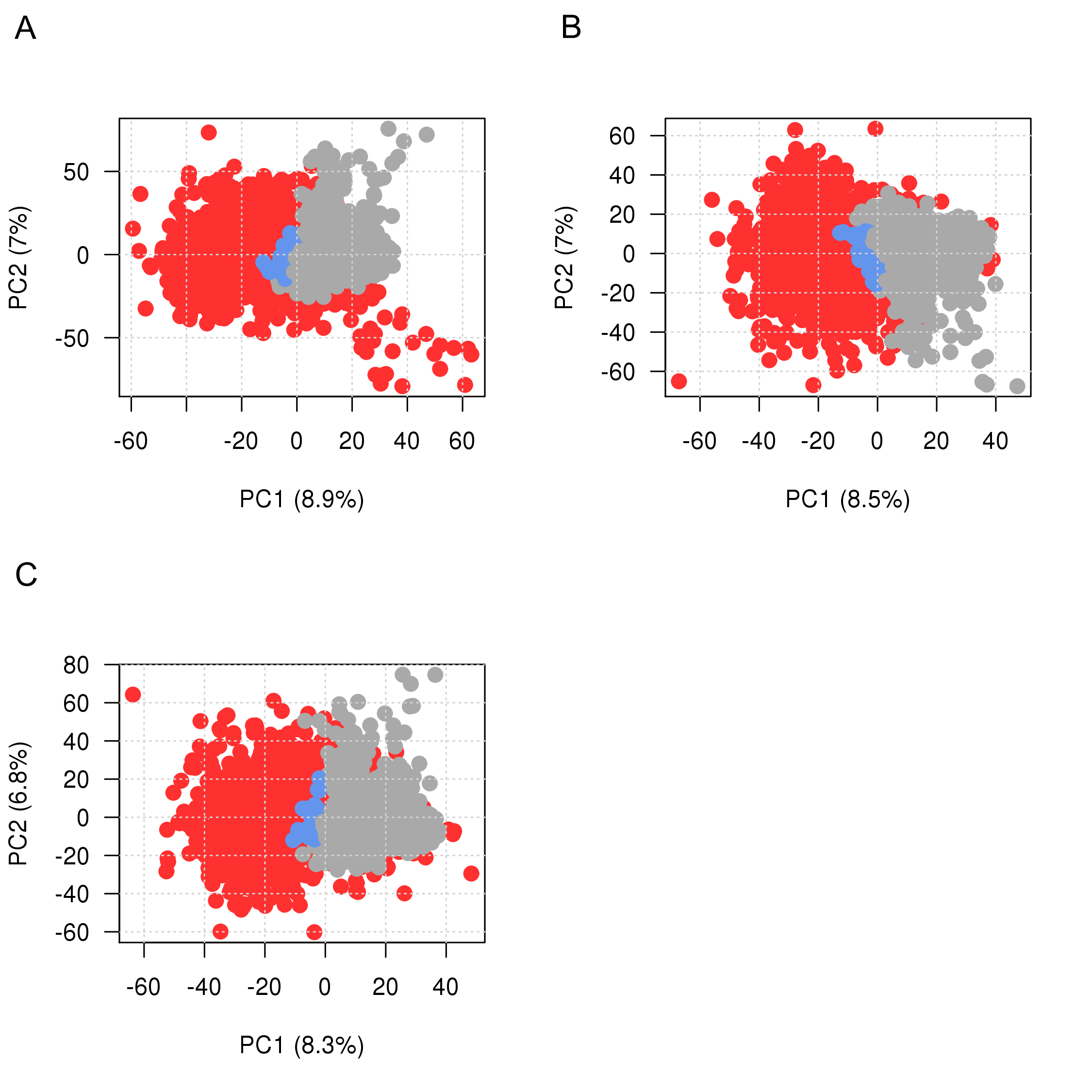

Supplement: S4 Fig — Different values for the parameter k, for the specification of an estimated number of unwanted factors of variation, were tested. Components of variance are visualized as PCA plots. Samples extracted and measured at baseline (BL) are marked red, repeated measures at 5-year follow up (BLFUrep) in blue and follow-up (FU) samples in grey. Batch correction based on ReplicateRUV with A: k = 2, B: k = 5 and C: k = 33 was performed. (TIF) [file pone.0156594.s004.tif]

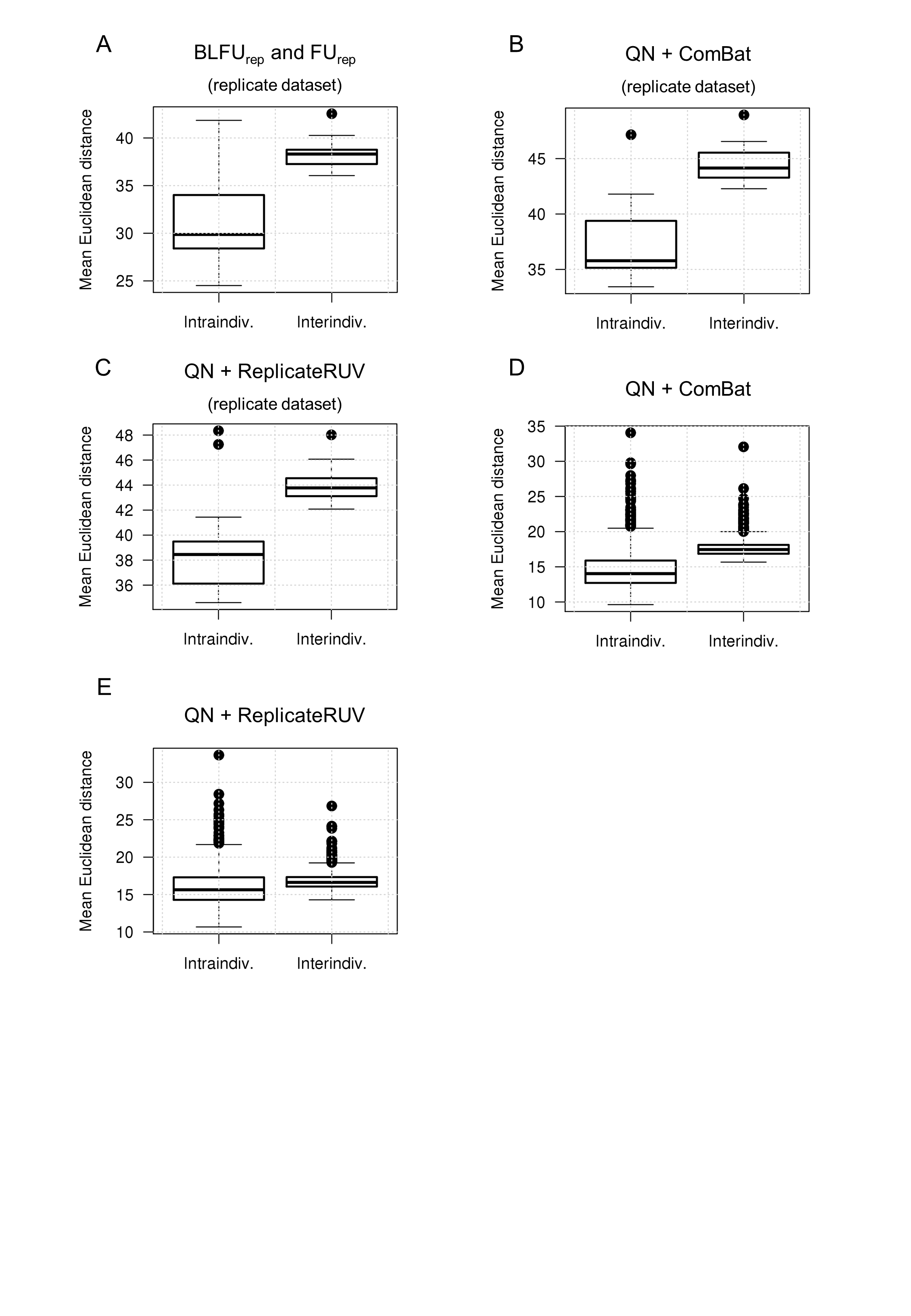

Supplement: S5 Fig — Intra-individual variation between time points was calculated by pairwise Euclidean distances between BL and FU for each individual. Inter-individual variation is specified by the mean Euclidean distance between one individual and all other individuals. A-C: replicate data set. A: Quantile normalized data from BLFUrep and FUrep samples—measured within one batch—mainly reflect biological differences and results are thus used as a reference. In contrast, observed differences between BLrep and FUrep include batch effects and biological variation between time points. A batch effect removal strategy that retains biological variation should therefore result in distributions comparable to A. Mean Euclidean distances are shown for batch effect removal by B: quantile normalization (QN) plus ComBat and C: QN followed by ReplicateRUV. The comparison of batch effect removal in the entire dataset by D: QN plus ComBat and E: QN plus ReplicateRUV indicates that QN followed by ComBat achieved the best results. (TIF) [file pone.0156594.s005.tif]

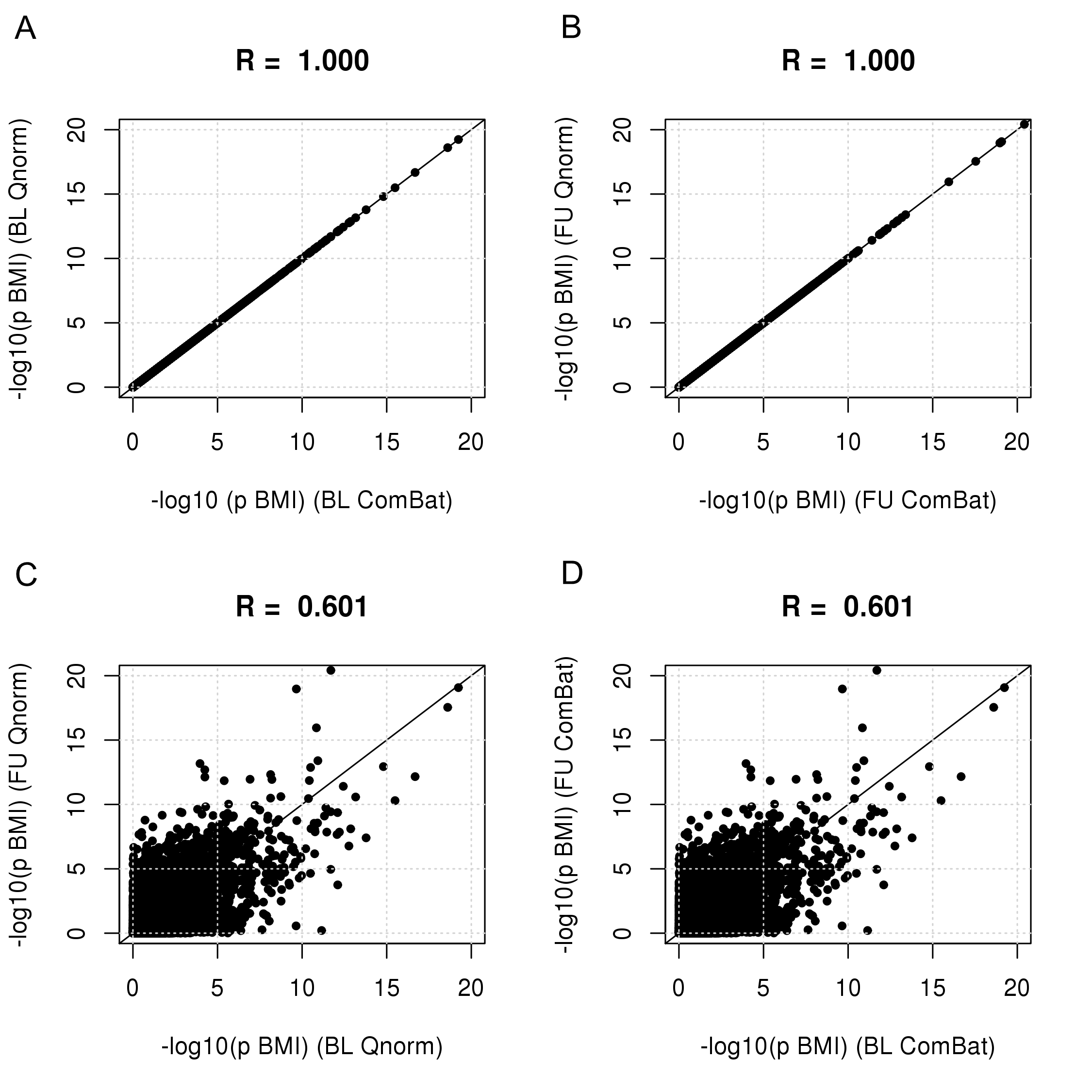

Supplement: S6 Fig — To assess whether biological sources of variability were maintained after batch effect removal, associations between each probe and body mass index (BMI) were calculated using linear mixed models within each batch containing 1092 samples before and after applying ComBat. A, B: For each probe, we plotted the p-values from ComBat corrected data on the x-axis and the quantile-normalized but uncorrected on the y-axis. BMI p-values were almost identical between corrected and uncorrected datasets in A: BL samples and B: FU samples. C.D: The BMI p-values from BL samples was plotted against the p-values observed in FU samples after C: quantile normalization and D: quantile normalization followed by ComBat. (TIF) [file pone.0156594.s006.tif]
