# Supplementary material for: Removing Batch Effects from Longitudinal Gene Expression - Quantile Normalization Plus ComBat as Best Approach for Microarray Transcriptome Data
Source: PLoS One. 2016 Jun 7;11(6):e0156594. doi: 10.1371/journal.pone.0156594 (PMC4896498; doi:10.1371/journal.pone.0156594)
Supplement: S3 Fig — Bland-Altman plots were produced using microarray data from one GHS individual to evaluate agreement of repeated measures before batch effect removal. Expression differences between technical replicates hybridized at baseline and 5-year follow up were therefore plotted against the mean expression of both time points for each probe. Each dot represents one probe and dense clusters of probes are marked blue. The majority of probes had low expression values. In uncorrected data, large expression differences between baseline and follow up data could be observed. Those differences were strongly dependent on the mean expression. (PDF) [file pone.0156594.s003.pdf]

S2 Fig. Direct comparison of expression between batches.

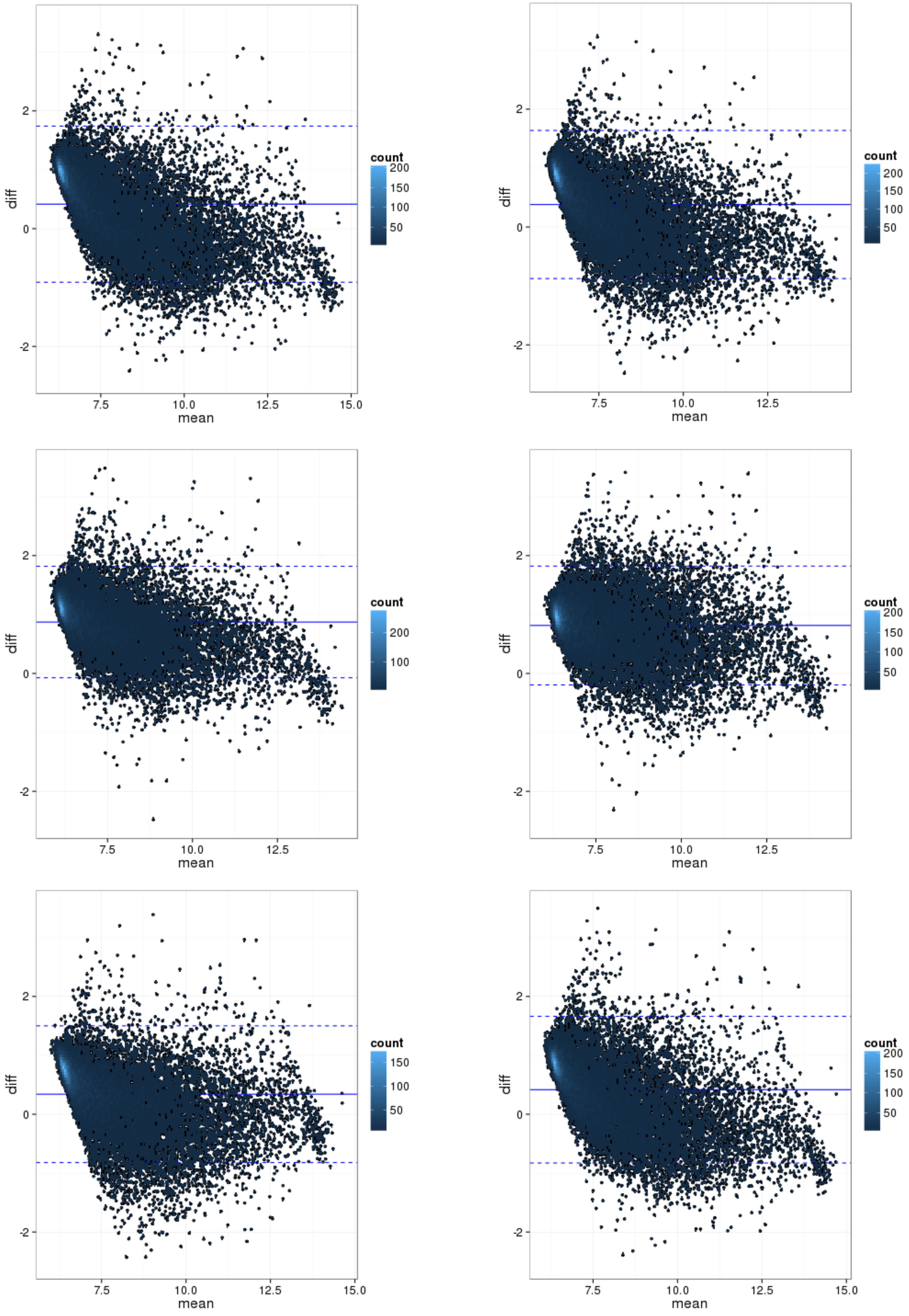

S2 Fig. Direct comparison of expression between batches.

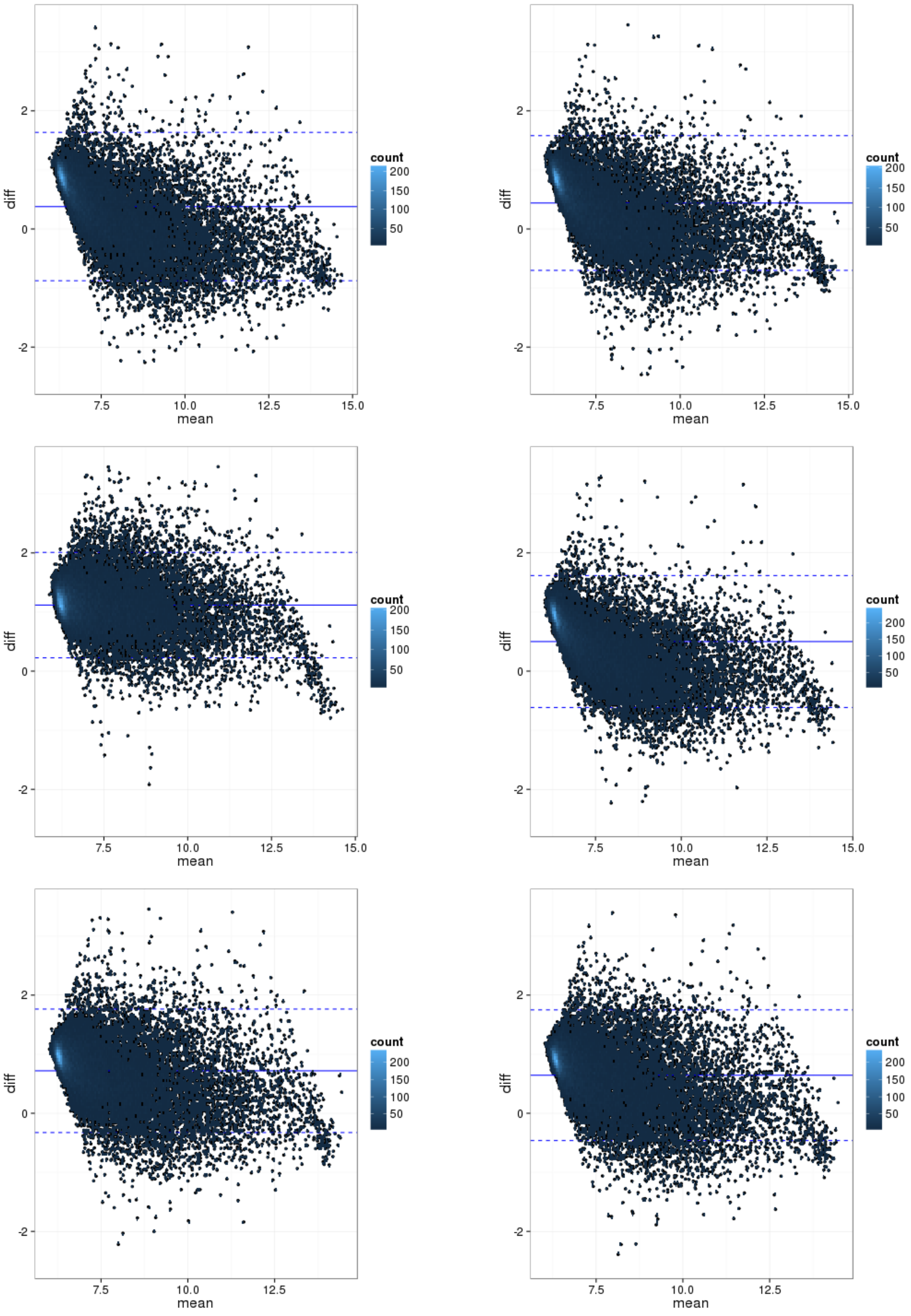

S2 Fig. Direct comparison of expression between batches.

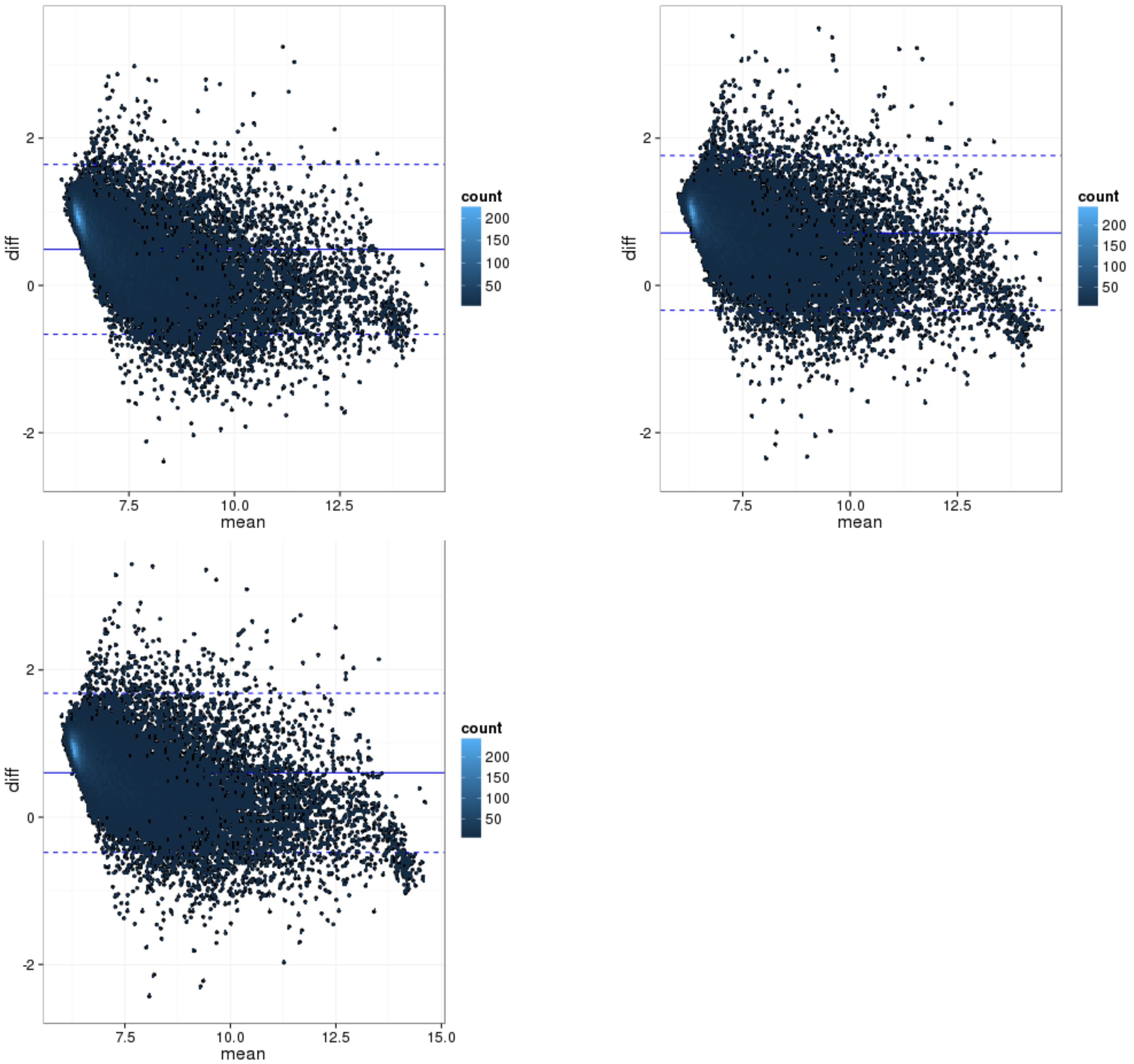

Direct comparison of expression between batches. Bland-Altman plots were produced using microarray data from one GHS individual to evaluate agreement of repeated measures before batch effect removal. Expression differences between technical replicates hybridized at baseline and 5-year follow up were therefore plotted against the mean expression of both time points for each probe. Each dot represents one probe and dense clusters of probes are marked blue. The majority of probes had low expression values. In uncorrected data, large expression differences between baseline and follow up data could be observed. Those differences were strongly dependent on the mean expression.
