# Supplementary material for: Removing Batch Effects from Longitudinal Gene Expression - Quantile Normalization Plus ComBat as Best Approach for Microarray Transcriptome Data
Source: PLoS One. 2016 Jun 7;11(6):e0156594. doi: 10.1371/journal.pone.0156594 (PMC4896498; doi:10.1371/journal.pone.0156594)
Supplement: S1 Material — (PDF) [file pone.0156594.s007.pdf]

S1 Material. Equations used for batch effect removal.

We assume that measured and log2-transformed expression values of gene *i* in sample *j* of batch X can be expressed in a general form as follows:

$$x_{i,j} = x'_{i,j} + b^X_{i,j} + \varepsilon^X_{i,j}$$

$x'_{i,j}$ represents the actual gene expression, $b^X_{i,j}$ the batch effect term and $\varepsilon^X_{i,j}$ reflects noise. We estimated $b^X_{i,j}$ by fitting linear regression models. Therefore, samples were assigned to either batch 1 or batch 2 representing baseline and follow-up measurements respectively. Subsequently, $b^X_{i,j}$ was subtracted from the observed gene expression $x_{i,j}$. For the linear mixed model based batch effect removal, a random variable was used to model sample pairs across batches.
